# Two-Dimensional Transition Metal Dichalcogenides and Their Charge Carrier Mobilities in Field-Effect Transistors

**DOI:** 10.1007/s40820-017-0152-6

**Published:** 2017-08-16

**Authors:** Sohail Ahmed, Jiabao Yi

**Affiliations:** School of Materials Science and Engineering, UNSW, Kensington, Sydney, 2052 Australia

**Keywords:** 2D materials, TMDC layers, Charge carrier mobility, Field-effect transistor, Heterostructure, Charge carrier scattering

## Abstract

Two-dimensional (2D) materials have attracted extensive interest due to their excellent electrical, thermal, mechanical, and optical properties. Graphene has been one of the most explored 2D materials. However, its zero band gap has limited its applications in electronic devices. Transition metal dichalcogenide (TMDC), another kind of 2D material, has a nonzero direct band gap (same charge carrier momentum in valence and conduction band) at monolayer state, promising for the efficient switching devices (e.g., field-effect transistors). This review mainly focuses on the recent advances in charge carrier mobility and the challenges to achieve high mobility in the electronic devices based on 2D-TMDC materials and also includes an introduction of 2D materials along with the synthesis techniques. Finally, this review describes the possible methodology and future prospective to enhance the charge carrier mobility for electronic devices.

## Introduction

Discovery of graphene has diverted the interest of researchers toward a new family of nanomaterials known as 2D materials. Unique properties of 2D materials have been widely utilized for diverse applications such as catalysis, supercapacitors, energy storage devices, and high-performance sensors. Besides graphene, transition metal dichalcogenides (TMDCs) and layered oxide materials are also parts of 2D materials family. 2D materials have shown promising properties in the application of electronic devices [1, 2]. Semiconductor behavior of 2D materials (e.g., MoS_2_) makes them promising materials for field-effect transistors (FETs). The FET fabricated from 2D materials will not only exhibit improved performance, in terms of fast processing rate and low power consumption, but also allow further reduction in device dimensions which is the need for the fabrication of next-generation electronic devices. In recent years, many efforts have been made to review the research on the synthesis, characterization of single- and few-layered 2D materials including their electronic, magnetic, optical and mechanical properties as well as applications [3–9]. Furthermore, some reviews focused on the utilization of 2D materials in a variety of applications such as flexible and transparent electronic, optoelectronic devices [10–15], energy conversion and storage [16, 17], hydrogen generation [18], and gas sensors [19]. It is well known that most 2D layered materials exist in a bulk state. These materials have layered structure and weak interlayer van der Waals force holding these layers together. Layered structure makes it possible to achieve monolayer or a few layers of 2D materials by mechanical exfoliation. On the other hand, in-plane atoms are connected with strong covalent bonds [1, 20]. Hence, monolayer 2D materials possess two-dimensional features in the lateral x–y direction and quantum confinement in the third dimension, which makes these materials unique from their bulk counterparts. One of the features is high carrier mobility, which is essential for high-speed transistors. For a high-performance transistor, good ohmic contact, higher carrier mobility, and appropriate band gap (~1 eV) are the basic requirements [12]. Graphene, the most extensively studied 2D material, has exhibited very high carrier mobility (~2 × 10^5^ cm^2^ V^−1^ s^−1^) at the temperature of 5 K [21]. However, it is not suitable for logic applications due to its zero band gap, resulting in very small on/off ratio (<10) at ambient temperature. In order to improve its on/off ratio, the opening of band gap has been proposed. However, engineering the band gap up to ~400 meV will lead to a decrease in the mobility to less than 200 cm^2^ V^−1^ s^−1^. Similarly, p-type device, based on the graphene nanoribbons, demonstrates a band gap opening due to the various edge (armchair or zigzag) structures resulting in very high on/off ratio of ~10^6^ and extremely low charge carrier mobility of ~100–200 cm^2^ V^−1^ s^−1^, as compared to other members of graphene family. However, these devices have shown the subthreshold slope (SS) of ~210 mV per decade, which is not desirable and ideal. It needs to be ~60 mV per decade at room temperature [22]. In contrast to graphene, TMDCs, such as MoS_2_ (1.8 eV), WS_2_ (2.1 eV), MoTe_2_ (1.1 eV), and WSe_2_ (1.7 eV), have desirable band gap [12, 23, 24]. MoS_2_ is one of the most promising materials for logic devices such as metal–oxide–semiconductor field-effect transistors (MOSFETs) due to its tunable band gap, high on/off ratio and relatively cheap price. The band gap of monolayer MoS_2_ has been reported to be 1.8 eV earlier. However, recent work demonstrates it has a direct band gap of 2.5 eV, while its bulk counterpart has an indirect band gap of 1.2 eV [25–28]. Being a relatively large band gap material, experimentally, MoS_2_ only shows low carrier mobility of ~1 cm^2^ V^−1^ s^−1^ without high-*k* dielectric gate material [29], whereas the mobility can reach ~150 cm^2^ V^−1^ s^−1^ at 300 K with HfO_2_ as the top-gate layer [30–33]. In addition, theoretical calculation based on density functional theory has indicated that the mobility of MoS_2_ can reach 400 cm^2^ V^−1^ s^−1^ [34] at room temperature. More recently, another type of 2D material, black phosphorus, has been predicted to have a hole mobility of 10,000–26,000 cm^2^ V^−1^ s^−1^ [35], and experimentally, a mobility of 1000 cm^2^ V^−1^ s^−1^ has been achieved [36], showing very promising potential for electronic devices [37].

In this review, we mainly focus on the recent development of the charge carrier mobility in 2D TMDC materials and the challenges for achieving high mobility as well as high current on/off ratio simultaneously, which are essential for 2D TMDC-based electronic devices. We also give an introduction of 2D materials (including TMDCs) and their synthesis using different approaches. Finally, this review describes the possible methodology and future prospective to enhance the charge carrier mobility for electronic devices.

## 2D Materials

Recent advancements in science and technology have unveiled the new prospects and put the mankind on the foundation of the newly developed field named as nanotechnology. This technology has enabled us to conduct research and work in the domain up to nanometer scale, resulting in the technologies which were never possible earlier. These technologies include cancer therapy based on nanoparticles, nanocomposites and innovative medicine, high-performance nanoelectronics, and highly sensitive sensors [38–41]. Evolution of nanotechnology has also introduced the distinguished class of low-dimensional systems such as zero-dimensional (0D, i.e., nanoparticles), one-dimensional (1D, i.e., nanowires), two-dimensional (2D, i.e., graphene), and three-dimensional (3D, i.e., bulk materials). The low-dimensional system plays an important role in classifying the nanomaterials, as the dimension of the material will not only define the atomic structure but also the properties of the nanomaterials [42].

In 2004, Geim and Novoselov [43] obtained a layered structure by mechanical exfoliation using scotch tape, which is later called graphene. The discovery of graphene has opened a new area of research—2D materials. These materials have shown many excellent properties widely used for energy, sensors, catalysis, electronic devices, spintronic devices as well as biomedical applications [3]. The discovery of graphene has triggered the research interest toward other two-dimensional materials, such as silicene, black phosphorus, transition metal dichalcogenides (TMDCs), and layered oxide materials. One of the most important features of these 2D materials is the high mobility of the carriers due to their quantum confinement in the third dimension, which is promising for the applications of electronic devices, such as transistors [1, 2].

As described previously, graphene has exhibited very high carrier mobility [21]. However, it is unsuitable for the applications in transistors since materials used to make transistors have to be able to switch current on and off to create logic circuits. Different from graphene, MoS_2_, one of the TMDCs, has a direct band gap in monolayer structure, which makes it possible to tune the carriers transport in an electronic device, thus realizing the device functions. Among the 2D materials, currently, TMDCs have attracted more and more interest due to their natural abundance and unique/diverse properties. The generalized chemical formula for TMDCs is MX_2_, where M represents the transition metal (typically Ti, Zr, Hf, Mo, W, Nb, Re, V, and Ni) of group 4–10 [26, 44] and X is a chalcogen (S, Te, and Se) [45]. Presently, more than 40 different TMDCs combinations have been reported [28, 46–48] and they have shown distinctive properties. In TMDCs, M (transition metal) layer sandwiched between two (02) X (chalcogen) atomic layers. Different atomic arrangements can generate the octahedral (tetragonal, T) and trigonal prismatic (hexagonal, H) structure of the 2D TMDCs. In H-phase TMDC, hexagonal symmetry can be observed from a top view and X-M-X arrangement is considered to be the monolayer, in which each M atom is covalently bonded to the six X atoms [49], whereas T-phase has a trigonal arrangement of the chalcogen (X) atoms on the top and shows the hexagonal structure of chalcogen atoms from a top view [50]. Among both, the 2H phase of 2D TMDCs is stable in air [51].The stability of mono or few layer TMDC is of the vital importance due to its layer dependent properties [52]. The conductivity of 1T MoS_2_ phase is 10^7^ times better than 2H MoS_2_ phase [53]. Due to the tunable band gap, quantum confinement and surface effects, monolayer 2D TMDCs (MoS_2_, WS_2_) exhibit strong photoluminescence (PL) and large exciton binding energy [54]. Besides the TMDCs, two-dimensional oxides have also been extensively investigated. Two-dimensional oxides include micas and layered oxides, such as MoO_3_ [55] and WO_3_ (micas) [56], TiO_2_, MnO_2_, V_2_O_5_, TaO_3_, and RuO_2_ [28, 57–61]. These oxides have also shown very promising properties for a variety of applications.

The family members of 2D graphene, TMDCs, and oxide materials are shown in Table 1. Gray color shows the monolayer fabricated by exfoliation. These 2D materials can be categorized in several ways in terms of their electrical, mechanical and transport properties, as they possess excellent and unique mechanical, thermal, optical, and electronic properties [62–65] which have the potential to replace current silicon-based semiconductor devices in the future.

**Table 1:**
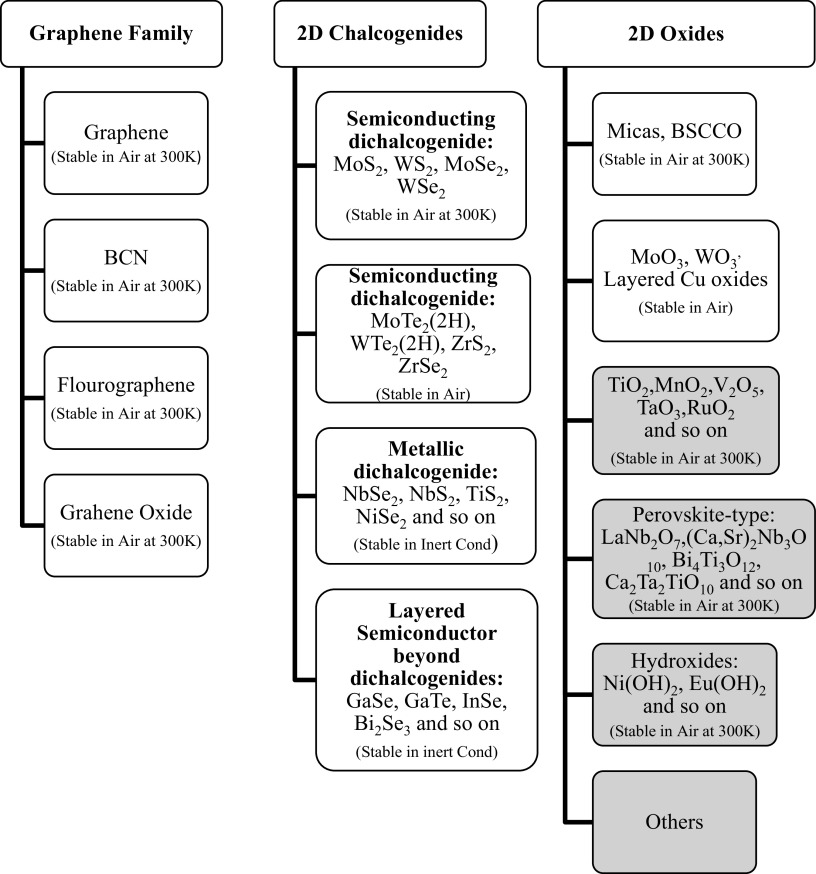
2D materials family [1, 9, 20, 29, 42, 66–70]

### Classification of 2D Materials

For a better understanding of properties and its applications, 2D materials can be categorized in following three classes.

#### Layered van der Waals Solids

The most common type of 2D material is the layered van der Waals solid which has strong in-plane covalent or ionic bond and weak interlayer van der Waals bonding. This weak out-of-plane bonding allows the extraction of mono or few layers of 2D materials from their bulk counterpart through mechanical or liquid exfoliation. The dimensions of these materials in the lateral direction are up to a few micrometers and are less than 1 nm in thickness. TMDCs, especially MoS_2_, MoSe_2_, and WS_2_, are the well-studied materials. Besides those materials described afore, presently there are more than 40 different combinations of 2D layered TMDCs (X-M-X) reported [46, 47]. The transition metal, presenting in TMDCs, occupies trigonal prismatic or octahedral coordinates and forms the hexagonal structure [71]. Besides TMDCs, there are some other members of layered van der Waals solids as well, such as Sb_2_Te_3_ [72], vanadium oxide [73], and h-BN [74] etc.

#### Layered Ionic Solids

In this type of 2D materials, charged polyhedral layer is present between two layers of halide or hydroxide layers and these layers are held together via electrostatic force between them. Ion exchange liquid exfoliation or ion intercalation can be used to exfoliate the mono or few layered 2D materials. Typical layered ionic materials exfoliated from ion exchange methods are KCa_2_Nb_3_O_10_ [75], RbLnTa_2_O_7_, K_2_Ln_2_Ti_3_O_10_ [75], and La_0.9_Eu_0.05_Nb_2_O_7_ [76].

#### Surface-Assisted Non-Layered Solids

This type of 2D nanostructure materials is synthesized by making the layers artificially stacked on a substrate with arbitrary angles [77]. The methods to synthesize these materials include epitaxial growth and chemical vapor deposition. Silicene is a typical example of this class. However, its instability at ambient condition is the real challenge to make it feasible for the application in electronic applications [78, 79]. Ge/Ag (100) and Ag(111) [80, 81], TiO_2_ [82] and MgO/Mo (001) [83], and Al_2_O_3_/SiO_2_ [84] are the examples of surface-assisted non-layered solids.

### Synthesis Techniques for 2D Materials

2D materials can be synthesized by following techniques:

#### Micromechanical Exfoliation

This process was first discovered by Geim and Novoselov in 2004 [43], and the monolayer graphene was achieved [85], in which parent bulk material was peeled off by micromechanical force of the scotch tape and placed on the surface of the photoresist. This cleavage is possible due to the weak interlayer van der Waals forces. Monolayers of NbSe_2_ [29], MoS_2_ [29, 86], and WS_2_ [87] are some of the examples obtained by this process besides graphene. Despite being the fast and cost-effective process, micromechanical exfoliation is not an industrial-level production of monolayer materials since most of the flakes are smaller than 20 μm in diameter. In addition to the monolayer, a few layers or very thick layers can also be achieved at the same time. On the other hand, because of the absence of chemical interaction, monolayer obtained by this process is highly crystalline and its structural integrity can be maintained. This monolayer has shown good stability at ambient conditions up to the months’ exposure [29, 88].

#### Liquid Exfoliation

In contrast to mechanical exfoliation, which is a low yield method, liquid exfoliation is capable of producing the mono or few layers of 2D materials at a large scale. Mono or few layers of Bi_2_Te_3_, TaSe_2_, MoSe_2_, MoTe_2_, BN, WS_2_, and MoS_2_ can be easily obtained by this method [1, 89–91]. This method can be categorized into four different forms such as oxidation, intercalation, ion exchange, and ultrasonic cleavage.

Graphene oxide can be synthesized with an oxidation method by treating graphite flakes with potassium permanganate or potassium chlorate and nitric acid or sulfuric acid or their mixture [92–94]. The addition and dispersion of epoxide functional groups or –OH in a polar solvent and subsequent sonication result in exfoliated graphene oxide. This method is also known as Hummers method or modified Hummer method [94, 95]. An oxidative form of liquid exfoliation is most suitable for layered materials possessing low reduction potential.

The intercalation technique is equally applicable to TMDCs and graphene. In this method, interlayer force decreases due to the intercalation of ionic or organic molecules, which results in a decrease in energy required for exfoliation [96, 97].

**Fig. 1:**
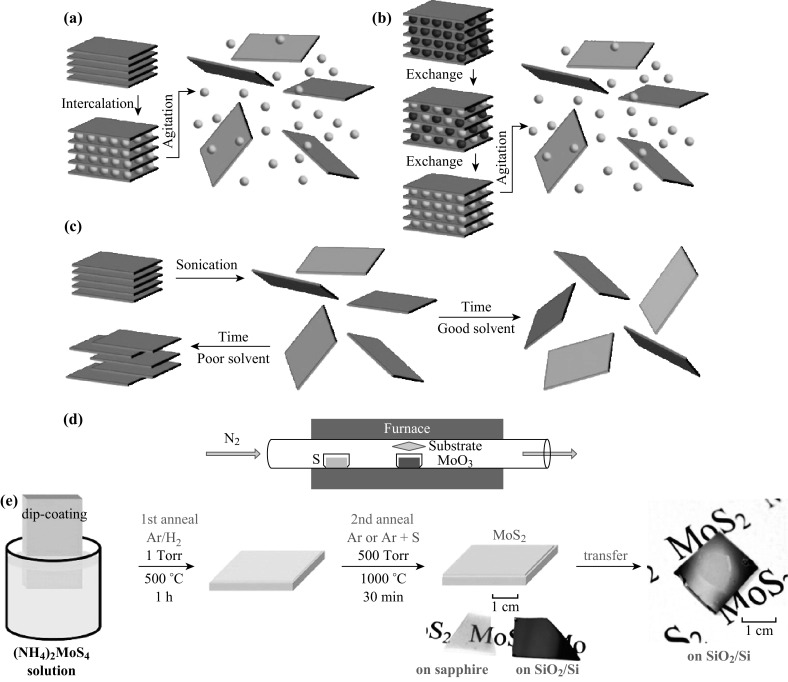
Schematic representation of the variation in liquid exfoliation process: **a** intercalation, **b** ion exchange, and **c** ultrasonic exfoliation. **a**–**c** Reprinted from Ref. [98] with permission from Copyright 2013, AAAS. **d** Schematic representation of the CVD process. **e** Schematic representation of large-area MoS_2_ nanosheet synthesis via dip and anneal technique. Reprinted with permission from Ref. [101], Copyright 2012, American Chemical Society

Another technique to obtain a single- or few-layered 2D material by liquid exfoliation is ultrasonic cleavage. In this technique, parent bulk material consisting of layers with weak interlayer bonding is ultrasonicated for 1–3 h after dispersion in a suitable solvent. In this process, cavitation bubbles are developed in the solvent due to high-energy ultrasonic waves [98]. The exfoliation of layers is possible due to the pressure released by the burst of cavitation bubbles. Subsequently, centrifugation is applied to separate the crystals. Figure 1a–c represents the schematic diagram of the above-mentioned forms of liquid exfoliation. Due to the complexity and destructive nature of liquid exfoliation method, layered 2D nanostructure material cleaved by mechanical exfoliation remains the favorite choice in the research community [9].

#### Chemical Vapor Deposition (CVD)

CVD is known as a bottom-up technique allowing the synthesis of 2D layers at a large scale with uniform thickness, which is promising for optoelectronics and electronic devices. Synthesis of MoS_2_ 2D nanostructured sheets by CVD on an oxidized silicon substrate is one of the recent developments. Generally, there are two ways for the fabrication of monolayer 2D materials. MoS_2_ as an example, first, in a so-called two-step bottom-up technique, a thin layer of metallic Mo is evaporated by an e-beam evaporation system. The Mo layer then reacts with sulfur vapors, generated by heating the elemental sulfur above its melting temperature. This reaction occurs at 750 °C leading to the formation of monolayered MoS_2_ [99]. This method can yield MoS_2_ with a thickness more than three layers. Second, a so-called one-step bottom-up technique is used for the synthesis of the atomically thick MoS_2_ nanosheets. In this approach, pure S and MoO_3_ powders are put in a CVD system. The mixture is heated to 650 °C. Then, the atomic layer of MoS_2_ will be grown on the Si substrate, which is covered with the rGO (reduced graphene oxide) for MoS_2_ layers growth. The rGO acts as a catalyst and the seed to enhance the growth of MoS_2_ layers [100], as shown in Fig. 1d. By this approach, MoS_2_ monolayer with a thickness of 0.72 nm has been obtained. Furthermore, large-area MoS_2_ nanostructured sheets can be obtained by dip coating of (NH_4_)_2_MoS_4_ (ammonium thiomolybdate) dissolved in DMF (dimethylformamide) on silicon or silicon dioxide substrate [100, 101]. Figure 1e shows the pictorial representation of this approach. After the (NH_4_)_2_MoS_4_ solution is dipped onto the substrate, the substrate is annealed under Ar/H_2_ atmosphere at 500 °C for 1 h. Then, the substrate is subsequently performed the second annealing at 1000 °C for 30 min under Ar or Ar^+^S atmosphere [101]. MoS_2_ 2D layer nanosheets are then obtained. X-ray diffraction (XRD), atomic force microscope (AFM), and transmission electron microscopy (TEM) analysis indicate that the layers grow and overlap each other without interlayer chemical bonding. Besides MoS_2_, other 2D materials such as TiS_2_ [102], VSe_2_ [103], WSe_2_ [104], WS_2_ [105], and MoSe_2_ [106] were also produced using CVD technique.

In addition, thermally decomposed (BN)_3_H_6_ (borazine) or (ClBNH)_3_ B-trichloroborazine can be used to fabricate the layered BN on transferred metal surfaces, e.g., Rh, Pd, Ru, Ni, and Pt, through an ultrahigh-vacuum CVD technique at a temperature above 700 °C [107–109].

#### Van der Waals Epitaxial Growth on a Substrate

This technique is similar to the CVD method. The only difference is that the substrate used in van der Waals epitaxial method also acts as a seed crystal. A variety of layered 2D nanostructured sheets can be synthesized by this method, such as MoS_2_, GaSe, h-BN, and hexagonal Si [107, 110–112].

Hexagonal Si is one of the examples achieved by this method. Si superstructures were deposited on Ag (001) substrate by heating the Si single crystal in ultrahigh vacuum using direct current. However, low-energy electron diffraction (LEED), scanning tunneling microscopy (STM), and XRD analysis indicated that there are two kinds of superstructures in the deposited layer [113]. Initially, a monolayer of Si nanostructure p (3 × 3) was formed. Subsequently, complex structure p (7 × 4) was observed. Direct current deposition formed the atomic thick Si nanoribbons on Ag (110) substrate. The atoms in Si nanoribbons were arranged in a honeycomb structure [114, 115]. Minor distortion was produced by the Si nanostructure on Ag substrate. Similar to that of hexagonal Si, monolayered Ge was also obtained by van der Waals epitaxial growth method on Ag (110) and Ag (111) substrates [116], whereas, due to the high solubility of Ge in Ag, the tetramers structure of Ge on Ag substrate leads to a larger distortion than that of Si [116].

Large-area 2D nanostructured materials, which are difficult to obtain via liquid and mechanical exfoliation, may be synthesized by van der Waals epitaxial growth method. However, the structure and properties of the 2D materials fabricated by this method are critically dependent on the orientation of the substrate and its chemistry. Moreover, in respect of future applications, this method is not suitable for the commercial applications, as it requires high vacuum and high temperature leading to high cost.

#### Hydrothermal Synthesis

Hydrothermal method is defined as the crystallization of substance from organic or aqueous solution at high vapor pressure and temperature. Due to extreme conditions, this method is only suitable for those precursors which can withstand these harsh conditions. 2D layered nanostructured materials have been successfully synthesized by this approach. Single-layered MoSe_2_ and MoS_2_ nanostructures were obtained through chemical reaction of Se/S with (NH_4_)_6_Mo_7_O_24_·4H_2_O (ammonium molybdite) in hydrazine monohydrate solution at a temperature of 150–180 °C for 48 h [117]. Furthermore, the single-step solvothermal reaction of hydrazine and (NH_4_)_2_MoS_4_ on GO, in C_3_H_7_NO (N, N-dimethylformamide) solution has produced the 3 to 10 layered MoS_2_ flakes [118].

Besides the synthesis of the MoS_2_ mono and few layered 2D nanostructures, other 2D materials have also been fabricated by the hydrothermal approach. Transition metal (groups IV and V) chalcogenides 2D nanostructures have been synthesized from metal chloride in oleylamine [119]. In this process, chalcogen sources such as elemental selenium, sulfur, or compound CS_2_ are employed. When sulfur is used as the source, irregular shape and poor crystallinity of TMDC is obtained which is mainly due to the highly reactive radical formation. In contrast, better crystallinity can be achieved when using elemental Se as the source [119]. When CS_2_ is employed initially, H_2_S is generated followed by its reaction with metal precursor, resulting in the formation of metal disulfide 2D layered crystals with variable lateral size in (001) plane. This growth is due to the low surface energy of this particular plane compared to (010) and (100) planes.

Besides the synthesis of TMDCs, this method has a potential to synthesize the 2D nanostructured sheets of hydroxides or metal oxides such as nanoribbons of VO_2_, which can be obtained by hydrothermal reduction in V_2_O_5_ in the presence of GOIt and is promising for applications in Li-ion batteries as a cathode [73]. Characterization techniques such as STM, X-ray photoelectron spectroscopy (XPS), and high-resolution transmission electron microscopy (HRTEM) have shown good composition control and crystal structure of 2D nanostructure materials synthesized by this approach.

### Properties and Applications of 2D Materials

The layered materials in which one dimension is restricted to a single layer are called 2D materials. In 2D materials, the increment in excitation energy leads to an increase in the density of states [120], resulting in different properties with different sizes and shapes of the quantum confined 2D materials. 2D materials exhibit dissimilar properties than their bulk counterpart and demonstrate the shape- and size-dependent properties, which make them suitable for a variety of nanoapplications [121].

2D layered nanomaterials have electron confinement and the layers are in close contact by van der Waals force, which results in a minimization of the interlayer interaction. Large surface-to-volume ratio allows altering the properties through surface treatments such as chemical functionalization [122]. Moreover, 2D materials can also be synthesized as dispersed nanoflakes, which can retain their properties similar to the monolayers. In addition, these nanoflakes can be mixed with other materials to form nanocomposites, which have been widely used for energy applications [89, 123, 124]. Highly conductive nature coupled with large surface area, excellent chemical stability, and flexibility make 2D materials as suitable candidates for the applications of energy storage and conversion [16]. 2D materials can also be utilized in fuel cells due to their photocatalytic properties [125]. Moreover, anodes made from graphene have shown enhanced cyclic lithium storage capacity (specific capacity of 460 mAh g^−1^), which can be utilized in the flexible battery devices [126–128]. 2D materials have also shown promising properties for supercapacitors. Recently, supercapacitors made from multilayered reduced graphene oxide have demonstrated a high specific capacity of 394 µF cm^−2^ [129].

Graphene has given rise to high charge carrier mobility, chiral properties of the mobile carriers, and high thermal conductivity [3]. Charge carriers in graphene can be well described by Dirac equation rather than the Schrödinger equation [130, 131]. It is well known that graphene is a gapless semiconductor. High symmetry of honeycomb lattice protects the zero gap of single-layer crystal. However, the band gap of bilayer graphene can reach up to 250 meV by the application of a transverse electric field and the band gap is tunable by an electric field [132]. Besides electric field effect, strain can also alter the low-energy band structure of layered 2D materials. For example, the band gap of a single- and bilayered MoS_2_ decreases linearly with strain at the rate of ~45 and ~120 meV/%, respectively [133, 134]. Furthermore, magnetic field also has a strong impact on electronic properties of 2D materials [43, 135, 136]. Charge carrier sign of 2D nanostructure materials can be modified by the application of electrical field allowing its utilization in high-mobility p–n junction transistors and complementary metal oxide semiconductor (CMOS) technology. Defect-free high-quality crystal up to micron scale [137] is the main reason for achieving high charge carrier mobility due to scattering free movement of electrons [138]. Therefore, 2D materials are very popular in the design of nanoelectronic devices such as field-effect transistors (FET) due to their high charge carrier mobility, high on/off current ratios, and low power consumption. FET composed of pristine graphene exhibits very high electron mobility (200,000 cm^2^ V^−1^ s^−1^) [21] but lacks in on/off ratio. In contrast, some 2D materials such as MoS_2_ (TMDC) exhibit variable band gap (1.2 eV, indirect to 2.5 eV, direct from bulk to a single layer) [26–28, 139]. Indirect-to-direct transition of band gap from bulk to the single-layered 2D material is due to the upshift of the indirect band gap induced by strong quantum confinement effect in a single layer [26]. This intrinsic large band gap of such single-layered 2D material gives rise to 10^8^ on/off ratio and ~150 cm^2^ V^−1^ s^−1^ electron mobility at 300 K for transistor application [30–33]. Furthermore, due to quantum limitations, channel thickness of <5 nm cannot be achieved in Si-based devices, as it reduces the carrier mobility significantly due to scattering generated by surface roughness [140]. Thus, two-dimensionality of TMDCs gives the advantage of dangling bonds free, fully terminated surface [13]. In comparison with Si transistors, it demonstrates 10^5^ times less power consumption [30]. Similar to TMDC, 2D phosphorene has also shown promising results for transistor applications [141]. In addition, mechanical strains and electrical field both have significant influences on the band structure of 2D nanostructured materials, which makes them suitable candidates for sensor applications. High mobility results in highly sensitive conductivity (to electrostatic perturbation) of 2D materials due to the possible generation of carriers on the surface via photo (light)-effects [142]. This property makes these materials potential candidates for high-gain photodetector application such as optical communications, optoelectronic devices, and biomedical imaging. 2D materials can also be used as the nanogenerators [6] which convert the biomechanical energy, induced by the human body motion, into the electrical signal. This property makes them promising for biosensor and body implanted device applications [6, 143]. 2D materials can absorb a large range of the electromagnetic spectrum (infrared to ultraviolet) [144] allowing their utilization in high-performance photonics and optics [145]. Atomic layer graphene can be used for ultrafast photonics application due to its wavelength independent ultrafast saturable absorption [146]. 2D topological insulators (e.g., Bi_2_Se_3_) have the potential to be utilized for nonlinear optics applications at high-power regime (low absorption and high nonlinear phase) [147]. Few-layered 2D TMDC materials or MoS_2_ nanoplatelets possess nonlinear optical properties and can potentially be used in laser photonic devices [148]. Black phosphorus is also a promising nonlinear optical material. Thin films of black phosphorus can be used in developing ultrafast photonic devices [149]. 2D materials have very low absorbance value (<10%) and high conductivity, making them suitable for flexible and transparent electronic applications such as liquid crystal devices and solar cells [150, 151].

Along with the extraordinary electrical and photonic properties, 2D materials also display excellent mechanical properties. The 2D material, such as graphene, is very flexible and has demonstrated 200 times higher breaking strength than steel [152]. Hence, it can be used to reinforce the polymers. Furthermore, its membrane can also be used in nonlinear electromechanical systems due to its extraordinary flexibility and ultrathin nature [153].

## Field-Effect Transistors

### Introduction

**Fig. 2:**
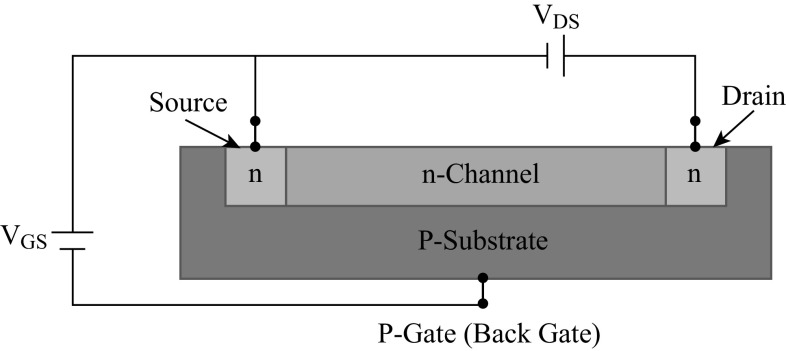
Idealized N-channel junction FET

In general, the transistor is a device which controls the flow of the electrical charge carriers across a semiconductor material by which it is fabricated. Field-effect transistors (FETs) are the particular class of transistors in which semiconductor material is used as a channel. The current carrier density and conductivity of a semiconductor channel are controlled by an applied voltage resulted from the regulated current flow passing through it by a supply. Both ends of the channel connected to the input supply are denoted as the source and drain terminals. The terminal which is responsible for controlling the conductivity and current flow through the channel, upon the application of the potential, is known as a gate terminal, as shown in Fig. 2.

**Fig. 3:**
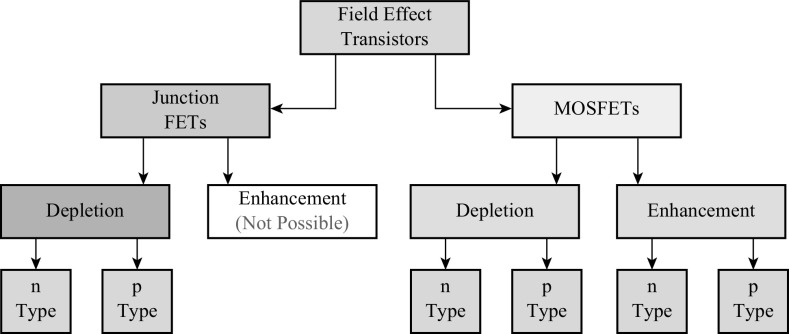
FET tree

The basic principal of the FET is not new and was first described by J.E. Lilienfeld in a patent of 1925. The development of electronic devices was made possible after the theoretical description of the concept related to FET, given by Shockley in 1952 [154]. The general field-effect transistors can be categorized into two major classes, junction FET (JFET) and metal oxide semiconductor FET (MOSFET), also known as an insulated gate FET (IGFET), as shown in Fig. 3. In the era of 1970–1980, the invention of a MOSFET has led to the revolution of the electronic circuits and the development of the microprocessors, resulting in the powerful portable calculators and computers. FET has a variety of applications such as protection devices, amplifiers, switches, current limiters, oscillators, mixers, and voltage-controlled resistors.

### Potential of 2D Materials in FET

Developments and advancements in materials have led to the exponential decrease in dimensions of the metal oxide semiconductor FET for over four past decades. This shrinking of size is the evidence of Moor’s law prediction. These advancements not only scale down the size of silicon-based MOSFET but also enable it to perform even faster compared to its ancestors. However, this miniaturization and fast processing are possible at the cost of high-power consumption. This high-power requirement is due to the short-channel effect which arises in the MOSFET having a dimension less than 50 nm and makes the gate control weaken over the channel current. This results in disability of the gate to completely switch “off” the current through the channel [155]. This flow of unwanted leakage current, at the “switch off” state of MOSFET device, gives rise to the high-power consumption and overheating problems. Further miniaturization of MOSFETs has been restricted due to the aforesaid challenges. To mitigate these complications and have better gate control to completely prevent the current flow through the device at “off” state, different approaches such as multiple gates [155] and high-k dielectric gate layer [156] have been investigated. However, tunneling field-effect transistor (TFET), another type of FET, in which carrier transport is due to the inter-band tunneling, can also show high I_on_/I_off_ with a low supply voltage which is attributed to the subthreshold slope (SS) <60 mV per decade [157, 158]. The potential of TFETs was first realized through the theoretical simulations [159, 160]. In addition, the research community is also looking for the alternate materials to complement or replace silicon technology. This brings the focus of researchers toward atomically thick mono or few layered 2D materials having unique characteristics appropriate for the future electronic devices, such as high mobility, transparency, and flexibility [161, 162]. The diverse properties of 2D materials, such as atomically thick body, robust nature, quantum confinement effect, high mobility, high switching efficiency, and tunable band gap, may result in the further scaling down of the device dimensions coupled with comparatively reduced short-channel effects [12, 21, 26, 30, 139]. In 2013, theoretical study, based on quantum transport simulations, shows promising results for reduction in channel length to ~10 nm [163]. Recently, experimental results, of U-shape MoS_2_ FET with 10 nm channel length, have demonstrated excellent short-channel behaviors [164].

As described previously, 2D materials such as graphene and TMDC were fabricated from their bulk form due to the layered structure, which is held by a weak van der Waals force [1, 20] although good ohmic contact, higher carrier mobility, and band gap (~1 eV) are the basic requirements for any material to be used in logic applications such as MOSFET, cost-effective large-scale synthesis of the material, and its compatibility with CMOS technology are also of great importance [165]. Moreover, 2D TMDCs also possess large relative effective mass for electrons (~0.5) and holes (~0.66) compared to Si (~0.29) [166] which results in reduced source–drain tunneling component in case of TFETs [167]. Researchers are still working hard to search new high-performance 2D materials or improve the current 2D materials for the requirements. In the past decade, significant progress has been achieved in the field of 2D layered materials for the state-of-the-art electronic nanodevices. However, there is still no 2D material meeting all the requirements for the high-performance 2D-based electronic device. For example, graphene shows very high mobility [21] but lacks in switching efficiency (<10) at ambient temperature. On the other hand, MoS_2_ has high I_on_/I_off_ ratio (~108) [30] due to its relatively large band gap [26–28, 139] but has low electron mobility [30–32] compared to graphene. Many research works have shown that the number of the layers has a profound influence on the performance of electronic devices and the properties of layers can also be affected by the substrate or another 2D layered material coupled with them. Therefore, to form a heterostructure from two kinds of 2D materials, such as graphene and MoS_2_, may be employed to achieve and I_on_/I_off_ ratio simultaneously [4, 168]. Along with the aforesaid essential properties, thermal conductivity and heat dissipation are also of vital importance for the realization and thermal management of the high-quality electronic device. The theoretical and experimental studies of the thermal behavior of 2D TMDCs, despite being the promising materials for FET applications, are still limited as compared to graphene and h-BN [169–173]. However, thermal properties of monolayer MoS_2_ (2D TMDC) differ from one-atom thin graphene layer due to its sandwich structure [174]. The thermal conductivity is mainly dominated by the phonons contribution rather than electrons [174]. Recently, theoretical investigations revealed the thermal conductivity of MoS_2_ is 1.35 [175], 6 [176] or 23.2 W m^−1^ K^−1^ [174] in different reports, which is lower in the magnitude than that of graphene. Moreover, experimental studies of MoS_2_ with a few layers have corroborated the thermal conductivity of 1.59 [177] and 52 W m^−1^ K^−1^ [178] at room temperature. Complete understanding of the thermal properties, of MoS_2_ (TMDCs), is crucial for the future state-of-the-art electronic device applications.

Along with the experimental research, semiconductor-based FET modeling and simulation serve as a bridge between manufacturers and designers [179, 180] and provide essential tools to explore the fundamental properties of 2D TMDCs for the device applications [181]. Various softwares (such as PHILIPAC, SLIC, and SPICE) are available to model and investigate the devices [182, 183]. These compact models for TMDC-based FETs are vital to study the device behaviors with computational efficiency and accuracy without loss of the physical insights [184]. Before the industrial-level production of the integrated circuits of FET based on TMDCs, the study of devices using the compact model is of prime importance. Compact model for analyzing these devices differs from Si-based devices because of the Fermi–Dirac statistics and density of states’ effects on the capacitance [185–187]. Recently, many researchers have shown interest in developing the compact model for TMDC-based FETs. Each compact model has its own significance. For example, one model is based on capacitive network, which considers drift component but ignores trap effects [188]. Whereas, another model has more focus on the subthreshold region of the device [189]. One of the models is developed to simplify the current and surface potential calculations with the help of the Boltzmann statistics [190]. Later on, the model is developed, for double-gate FETs, based on Fermi–Dirac statistics which involves implicit equations but excludes the trap effects [187]. The research work in [191] demonstrates the compact model, which also considered the Fermi–Dirac statistics with drift–diffusion transport. These models were developed to explore the behavior of the TMDC channel-based FETs.

### Applications of 2D TMDC Materials in MOSFET

**Fig. 4:**
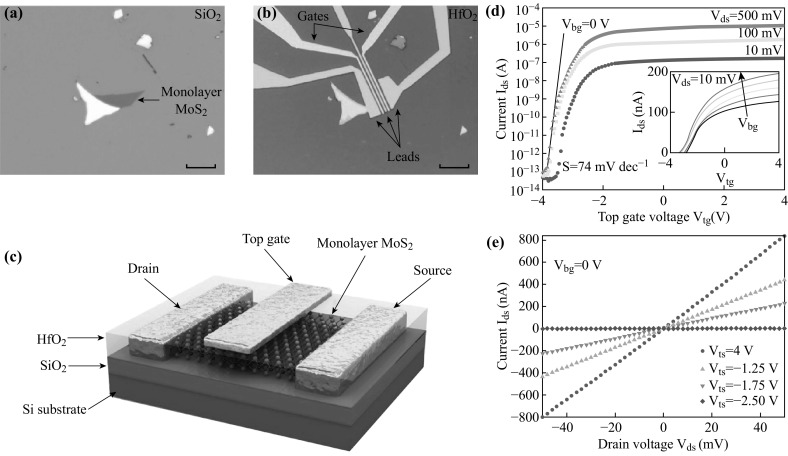
**a**–**c** Preparation process of the single-layer MoS_2_ transistor. **d** and **e** the gate control of single-layer MoS_2_ transistor. Reprinted with permission from Ref. [30], Copyright 2011, Nature Publishing Group

Metal oxide semiconductor field-effect transistors (MOSFETs) currently are the main components of any logic devices, and each digital circuit is composed of many logic gates. Any material chosen to be used in the fabrication of logic devices needs to satisfy some basic requirements such as low conductance at off condition to minimize the standby power consumption, high on/off switching ratio from 10^4^ to 10^7^, and higher mobility of charge carrier for swift operation [192]. Large band gaps (1.2–2.5 eV) of bulk, few- or single-layered MoS_2_ make it a suitable candidate to provide high switching ratio with low off-state power consumption [27, 28, 30]. Because of high switching ratio and large band gap, MoS_2_ being the potential candidate has been extensively studied for its application in the logic device. In this review, we will focus on MoS_2_-based FET as an example. Initially, low charge carrier mobility of 0.5–3 cm^2^ V^−1^ s^−1^, which is not sufficient for logic devices, was reported [29]. Subsequently, mechanically exfoliated nanopatches of MoS_2_ exhibited a high on/off ratio >10^5^ and charge carrier mobility in tens of cm^2^ V^−1^ s^−1^ [193]. High switching on/off ratio of 10^8^, charge carrier mobility higher than ~60 cm^2^ V^−1^ s^−1^, and subthreshold swing of 74 mV per decade (increase in gate voltage required to change the drain current by one decade. Here decade represents the 10 times increment in drain current) were reported in top-gated MoS_2_-based FET at room temperature [30–32]. Subthreshold swing gives the qualitative and quantitative analysis of I_on_/I_off_. A lower value of subthreshold swing for FET will result in higher I_on_/I_off_ and better switching behavior. At room temperature, the ideal subthreshold value is 60 mV per decade [13]. Similarly, monolayered MoS_2_ FET has also shown on/off ratio of 10^8^ and charge carrier mobility of ~150 cm^2^ V^−1^ s^−1^ at 300 K once dielectric HfO_2_ was used as the gate layer [30–33, 194] (Fig. 4).

**Fig. 5:**
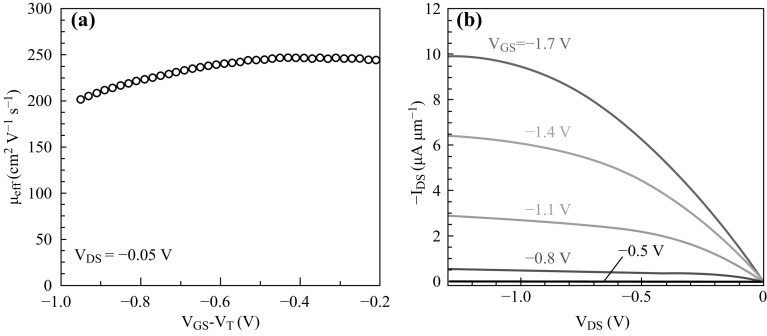
**a** Effective hole mobility as a function of gate overdrive and **b** output characteristics of the *p-*type monolayer WSe_2_ device. Reprinted with permission from Ref. [168], Copyright 2012, American Chemical Society

MoS_2_ showed low power consumption when high-*k* dielectric gate layer in top-gated configuration was used. Besides the high performance in FET, photoresponsivity in the range of 100–680 nm and ultrasensitivity of 880 A W^−1^ at a wavelength of 561 nm was observed in monolayered MoS_2_ photodetectors [195]. The excellent optical properties of MoS_2_ make it a potential material for the applications in video recording, optoelectronics, light sensing, and biomedical imaging applications [195–197]. Besides MoS_2_, WSe_2_, a p-type TMDC, is also equipped with the set of properties required for the device applications, such as considerable bulk indirect and monolayered direct band gap (1.2–1.65 eV) [198, 199]. FET based on bulk WSe_2_ exhibited promising room-temperature hole (charge carrier) mobility of ~500 cm^2^ V^−1^ s^−1^ [200]. However, it lacked in on/off current ratio (>10). Bulk nature of WSe_2_ results into large “OFF” state current which is not desirable for the efficient FET applications. In contrast, mechanically exfoliated single-layered WSe_2_-based p-type FET has shown the hole mobility of ~250 cm^2^ V^−1^ s^−1^ with the enhanced on/off current ratio (>10^6^) and ideal subthreshold slope of ~60 mV per decade, as shown in Fig. 5 [199]. Moreover, p-type FET fabricated from CVD grown monolayer WSe_2_ has demonstrated the hole mobility of ~90 cm^2^ V^−1^ s^−1^ with an appropriate on/off current ratio of 10^5^ [198, 201]. Similarly, MoTe_2_-based FETs show the ambipolar response. Both n- and p-type material can be synthesized by controlling the growth method [202]. Theoretical calculations have depicted the room-temperature mobility of MoTe_2_-based FET is of ~200 cm^2^ V^−1^ s^−1^ [203]. However, the recent experimental works for three-layered MoTe_2_ device with Ti contacts have presented charge carrier mobility of 7 × 10^−2^ and 2 × 10^−2^ cm^2^ V^−1^ s^−1^ for holes and electrons, respectively [204, 205], whereas, in another work, a similar MoTe_2_ device with Au contacts has shown improved mobility of 16.5 cm^2^ V^−1^ s^−1^ with on/off current of 10^7^ [204].

Regarding the current research of TMDCs on FET, charge carrier mobility is the main focus. The impurities presented in mono or few layered MoS_2_ material lead to screening effect, which influences the dielectric environment on charge carrier mobility. The large dielectric constant of the gate layer will increase the capacitive coupling between top gate and back gate which in return stimulates the charge carrier mobility by 10–50 times [194]. If only silicon/silicon dioxide is used as the substrate without high-k dielectric gate layer in mono or few layered MoS_2_-based FET, charge carrier mobility of about 10 cm^2^ V^−1^ s^−1^ can be obtained at room temperature. Hence, the low mobility is due to the poor interface between Si/SiO_2_ and MoS_2_ [206, 207]. This interface problem including surface defects, the concentration of charged impurities, local charge distribution, and trapped charges in the substrate results in coulomb scattering and low charge carrier mobility. The use of high-k dielectric materials, such as hafnium dioxide (HfO_2_) [30–32] or alumina (Al_2_O_3_) [208] as top gate, has increased the charge carrier mobility to ~150 and 500 cm^2^ V^−1^ s^−1^, for monolayer and bulk MoS_2_, respectively, due to the screening effect. Besides the influences of high-*k* dielectric gate layer and channel material (MoS_2_) characteristics, charge carrier mobility has been underestimated due to the presence of Schottky barriers between metallic contacts and MoS_2_ single or few layers [194, 209]. The reduction in resistance between the contacts and thinning of Schottky barrier results in an enhancement of charge carrier mobility from 100 to 220 cm^2^ V^−1^ s^−1^ [210]. Theoretical calculations based on density function theory suggested that the carrier mobility of MoS_2_ at room temperature could reach 400 cm^2^ V^−1^ s^−1^ [34]. These results suggest that MoS_2_ is a promising material for future electronics.

## Current Challenges in 2D Materials Device Application

Experimentally demonstrated charge carrier mobility of the mono or few layered MoS_2_ is much lower than that of theoretical prediction of 410 cm^2^ V^−1^ s^−1^ [34]. 2D materials possess large surface area making charge carrier mobility more sensitive to the external (e.g., trapped charged impurities, interface quality, and adsorbates from ambient air) and internal (lattice defects) factors [206, 207]. An experimental result of charge carrier mobility depends on the measurement condition/method and sample quality. Other factors including channel thickness, measuring temperature, annealing, Schottky barrier, dielectric environment, and carrier density (n) play an important role in achieving high charge carrier mobility devices.

The influence of channel thickness on carrier mobility was ascertained when mechanically exfoliated MoS_2_ and NbSe_2_ demonstrated 0.5–3.0 cm^2^ V^−1^ s^−1^ charge carrier mobility, which is lower than their bulk counterparts [29]. Lee et al. [211] also showed the reduction in carrier mobility with the decrease in thickness placed on different substrates (SiO_2_ and h-BN). One- to 5-layered MoS_2_ displayed charge carrier mobility varying from 10 to 50 cm^2^ V^−1^ s^−1^ [212]. The possible reasons for the thickness dependence are coulomb scattering and the Schottky barrier between channel and electrode contacts [213]. Moreover, in recent work, Hall mobility of ~1000 and ~34,000 cm^2^ V^−1^ s^−1^ is reported for single- and six (6)-layered encapsulated MoS_2_ devices, respectively, at a temperature below 5 K [214]. Just like the channel thickness, measuring or operating temperature has a significant effect on carrier mobility. High temperature (>100 K) enhances the lattice phonon scattering which tends to have an adverse effect on carrier mobility of 2D TMDCs. Consequently, the theoretically predicted charge carrier mobility of 410 cm^2^ V^−1^ s^−1^ at room temperature [34, 215], limited by phonon scattering, has never been achieved experimentally for single-layered MoS_2_ [216]. Similar behavior has also been reported for MoSe_2_ with a few layers [217]. At low temperatures, high field-effect mobility (~1000 and ~34,000 cm^2^ V^−1^ s^−1^) of single- and six-layered MoS_2_ has been reported [214, 216]. Moreover, charge carrier density (*n*) also plays a vital role in electronic properties of 2D TMDCs materials. MoS_2_ has shown electronic phase transition (metal to insulator) with an increase in charge carrier density [218, 219], whereas medium range charge carrier density is essential for the transistor applications. In this range, an increase in charge density may have two outcomes. First, high charge density is favorable for increasing carrier mobility by suppressing the interfacial impurity potential. Second, high carrier density tends to increase the carrier energy which may result in reducing carrier mobility [212].

Interfacial Schottky barrier, between the channel semiconductor and metal electrodes, reduces the charge carrier mobility by inducing resistance in carrier transfer. This unwanted effect can be minimized by employing four-terminal measurement method [220]. Formation of the Schottky barrier is due to the difference in energy levels between the semiconductor and electrode materials. Schottky barrier height is directly proportional to the energy-level difference of two coupled materials [221]. Barrier height can be reduced by making contact between *n-*type semiconductor and low work function metals or *p-*type semiconductor and high work function metal. Moreover, the Schottky barrier is tunable by varying the FET gate bias. Initially, this variation was considered to be the switching mechanism of FETs [222]. Later, the dependence of barrier height on channel thickness was reported. In thicker flakes, the barrier height is much smaller than that of monolayered 2D TMDCs materials [223]. It is due to the influence of band gap of semiconductor channel on the Schottky barrier. Thinner (<5 layers) MoS_2_ possesses larger band gap which results in higher barrier height and contact resistivity than bulk MoS_2_ [220]. Barrier height between Au and MoS_2_ varies from 0.3 to 0.6 eV with a decrease in the number of MoS_2_ layers from 5 to 1 [220]. Hence, charge carrier mobility can be improved by optimizing the metal/semiconductor contact. Furthermore, minimum electrode length needs to be identified in order to obtain better charge transferring efficiency [220]. Channel length (in appropriately annealed samples), however, does not affect the MoS_2_ device performance [220]. In addition, some studies have shown that the presence of ultrathin TiO_2_ [224] or MgO [225], between MoS_2_ and ferromagnetic metal, reduces interfacial Schottky barrier height.

Scattering in semiconductor channel is one of the reasons for the reduced charge carrier mobility. The scattering may be induced by lattice phonons due to the high-k dielectric environment, charged impurities, and interfacial phonons. At room temperature, bulk MoS_2_ has exhibited the electron mobility ranging from ~150 to 500 cm^2^ V^−1^ s^−1^ [30–33, 208]. However, if the monolayers of MoS_2_ fabricated by mechanical exfoliated are transferred on SiO_2_ to make a device, the carrier mobility then drops down to 0.1–10 cm^2^ V^−1^ s^−1^ [29]. High charged impurity density (*N*) gives rise to coulomb scattering which can be screened with the use of high-*k* dielectric material, leading to the increase in electron mobility at low temperatures [219]. In FETs made of 2D TMDC materials, charged impurities (coulomb) are present at the interface between dielectric and 2D channel. These charged impurities have a scattering potential which induces the scattering in 2D FETs [226–228]. The source of charged impurities includes chemical residues, adsorbates introduced during device fabrication, and contaminated surface, which results in reduced charge carrier mobility of the 2D TMDC-based FETs. Theoretical calculations and experimental results have demonstrated a high rate of coulomb scattering in the thinner channel with HfO_2_ or SiO_2_ as the gate layer [212], indicating that the top/bottom surface scattering is associated with channel thickness. As the thickness decreases, interaction distance between charge carriers and charged impurities shrinks due to the electrostatic equilibrium. This shrink in interaction distance results in high scattering potential and lower charge carrier mobility [212].

Charge carriers can be scattered by lattice phonons through potential deformation. Adjacent atoms move in the direction of acoustic phonons but opposite to the optical phonons. Phonon scattering is proportional to temperature and increases with the increase in temperature. Based on theoretical calculations (by the first principals calculations) of acoustic/polar phonon scattering and screening for single-layered MoS_2_, charge carrier mobility of ~410 cm^2^ V^−1^ s^−1^ has been reported [215, 229, 230]. However, these calculations did not cater for the effects of dielectric mismatch and free-carrier screening. Phonon scattering becomes dominant due to the presence of high-*k* dielectric material and leads to a drastic decrease in electron mobility at room temperature (300 K) [230]. This phenomenon tends to increase as the thickness of the semiconductor layer is decreased from bulk to monolayer. In TMDC materials like MoS_2_, polar nature of the chemical bonds gives rise to the dipole moments between anions and cations. Perturbation of dipole moment by polar phonons creates an electric field which is coupled with charge carrier, resulting in low charge carrier mobility. This phenomenon is called Frohlich interaction or polar optical phonon scattering [229]. Phonons can be excited by the charge carriers if the dielectric layer in FETs supports polar vibrational modes. These phonons are known as surface optical phonons or remote interface phonons. At room temperature, scattering due to remote interface phonons is more dominant in the high-k dielectric environment compared to low *k* dielectric gate layer [231, 232].

Besides coulomb and phonon scattering, structural defects also play a vital role in the degradation of charge carrier mobility. Structural defects include dislocation, vacancies, grain boundaries, impurities, and precipitation. In a low-quality sample, anion vacancies can act as a strong scattering source. Studies have shown that CVD grown or mechanically exfoliated MoS_2_ possesses high percentages (0.4%) of sulfur vacancy which affects the charge carrier mobility [233]. Vacancy repairing of CVD grown single-layer MoS_2_ by annealing can improve the charge carrier mobility up to 45 cm^2^ V^−1^ s^−1^ [234]. Vacancy scattering is independent of carrier density and weakly depends on the temperature and channel thickness as these parameters do not have any direct relation with the defect densities [235]. Besides vacancy defects, the presence of tilt grain boundaries may also degrade the charge carrier transport efficiency [236].

**Fig. 6:**
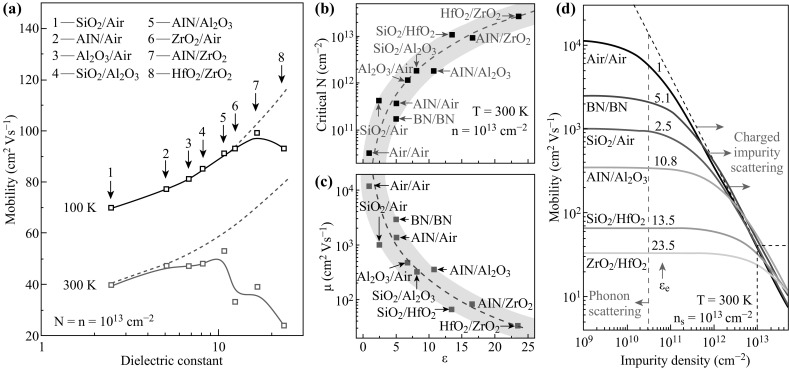
**a** Electron mobility as a function of dielectric constant. Dashed lines show the mobility without considering the SO phonons. **b** The critical impurity densities Ncr and **c** Room-temperature electron mobility in monolayer MoS_2_ surrounded by different dielectrics. **d** The room-temperature net electron mobility in monolayer MoS_**2**_ considering all kinds of scattering mechanisms as a function of impurity density (*N*). Reproduced from Ref. [230]

Electron or charge carrier density (*n*) is also one of the factors affecting the charge carrier mobility. Although electron mobility tends to increase with the increase in electron density, the effect is prominent after the effective screening of phonon scattering. As the dielectric value of *k* increases, electron mobility drastically decreases due to phonon scattering. Electrons presented in atomically thick 2D mono or few layer nanosheets excite the phonons in the nearby dielectric material (which supports polar vibrational modes) at room temperature. Figure 6a [230] demonstrates the effects of dielectric environment on charge carrier mobility at two different temperatures of 100 and 300 K. In both cases, electron density and charged impurity density are considered equal to 1013 cm^−2^. The solid line represents the combined effects of coulomb and phonon scattering on mobility, whereas the broken line shows the electron mobility while neglecting the effects of phonon scattering, which indicates a trend to achieve large mobility at high-k dielectric environment (*ε*_e_). In this case, mobility is entirely dependent upon the charged impurity density.

At low temperature, when the phonon scattering is inactive and negligible, electron mobility can be improved by decreasing the charged impurity density. If the charged impurities density is low, low *k* dielectric is required for coulomb screening, which will result in less phonon scattering at room temperature [230]. Figure 6b [230] shows the dielectric constant required against the critical value of charged impurity density *N*_cr_ at room temperature. If *N* > *N*_cr_, then the electron mobility highly depends upon *N* rather than phonon scattering and vice versa. Figure 6c [230] represents the charge carrier mobility of a monolayer MoS_2_ (phonon limited) at room temperature under different dielectric environment ranging from free suspension in the air to high-*k* dielectric HfO_2_. It can be inferred from the plot that the phonon determined mobility decreases with the increase in dielectric constant of the gate layer. Figure 6d [230] demonstrates the combined room-temperature electron mobility in monolayer MoS_2_ nanosheet against variable charged impurity densities under various dielectric environments considering all kinds of scattering mechanisms. The carrier density is fixed to be 1013 cm^−2^. From Fig. 6d, one can figure out that the electron mobility is weakly dependent on the dielectric environment at high impurity density (1013 cm^−2^), as shown in the dashed box in the bottom right corner.

## Future Prospective and Conclusion

Aforementioned challenges, regarding the utilization of 2D nanomaterials in device applications in connection with the charge carrier mobility, drive us not only to produce the high-quality (defect- and impurity-free) single-crystal mono or 2D nanosheets with a few layers but also to think about searching new materials. This path leads to two methodologies pertaining to the improvement of charge carrier mobility by the utilization of existing materials via special design, such as heterostructure technique or experimental study of some new materials, which is possible to have high carrier mobility.

For the first methodology, efforts need to be made to produce the high-quality and defect-free 2D nanostructure which contains a minimum level of charged impurity density. Mechanical exfoliation can produce high-quality TMDCs. However, the difficulty for large-scale synthesis with uniformity and reproducibility of samples are the main limitations of this process. CVD is a method which has the potential of producing the large-scale high-quality 2D TMDCs or graphene [237, 238]. In order to obtain the impurity-free high-quality 2D materials, the ultraclean substrate (flushing of the substrate with acetone and isopropanol and subsequently vacuum annealing at high temperature) and highly pure precursors need to be used. In addition, the synthesis parameters such as pressure, temperature, and growth time are needed to be optimized, which are all important to achieve large-scale, high-quality single-crystal 2D materials [237–239]. Proper procedures to avoid contamination during 2D material synthesis are also of importance. For example, the purging of Ar gas with a very high purity is required to eliminate any chance of impurity in the samples [240]. Thickness and number of layers of 2D material can be controlled by controlling the deposition parameters, such as heating temperature, deposition time, and chamber pressure [238, 241]. Furthermore, very recent developments have shown that adding a small amount of O_2_ in the Ar carrier gas can also suppress the nucleation and promote the synthesis of MoS_2_ with a large area and high quality [239]. In addition, device fabrication and encapsulation inside Ar-filled glove box will also help avoid device degradation, possibly due to the presence of chemical species (e.g., O_2_ and H_2_O) in ambient condition [242].

**Fig. 7:**
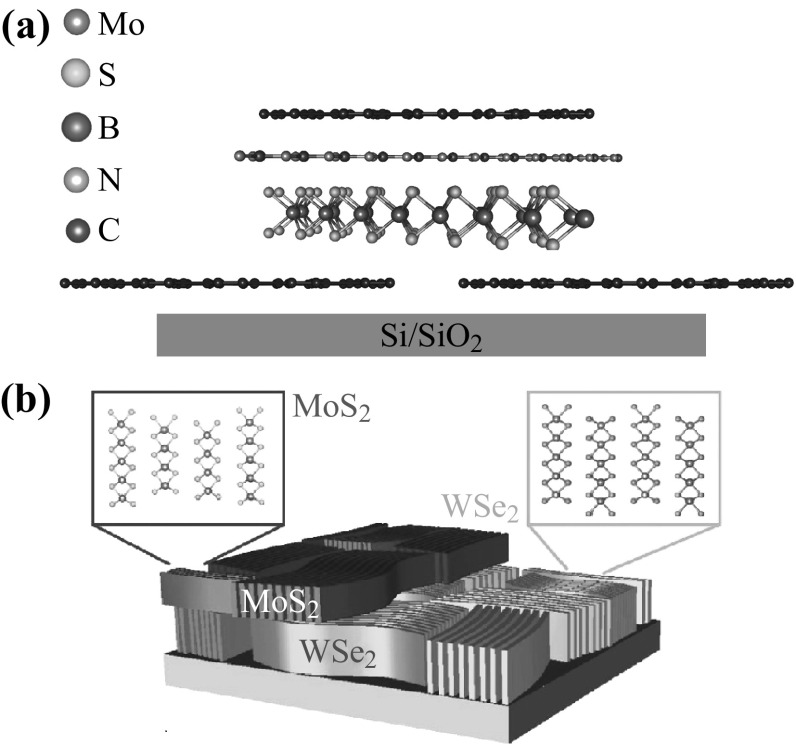
Heterostructure-based **a** FET composed of MoS_2_ (channel), h-BN (top-gate dielectric), and graphene (source, drain, and top-gate electrodes). Redrawn from Ref. [245] and **b** p–n junction fabricated from vertical stacked MoS_2_ (*n* type) and WSe_2_ (*p* type). Reprinted with permission from Ref. [247]. Copyright (2015) American Chemical Society

From the above discussions, charge carrier mobility in 2D materials is primarily dependent on the concentration of the charged impurities and then on the phonon scattering due to the dielectric environment at high temperatures. CVD method has the potential to generate high-quality large-scale single-crystal 2D nanosheets. MoS_2_, graphene, WS_2_, and MoTe_2_ high-quality monolayers have been extensively reported using CVD techniques. Recently, it has been observed that assembling different 2D materials into heterostructure may lead to the tuning of electronic properties [243, 244]. A band gap of 0.1 eV of MoS_2_ was achieved after a heterostructure composed of MoS_2_, and graphene was formed [243]. Furthermore, heterostructure-based FET composed of MoS_2_ (channel), h-BN (top-gate dielectric), and graphene (source, drain and top-gate electrodes) has displayed charge carrier mobility of ~33 cm^2^ V^−1^ s^−1^ and on/off current ratio of >10^6^ [214, 245] (Fig. 7a). Besides MoS_2_, TMDCs p–n junction (heterostructure) also demonstrates promising results. Heterostructure of TMDCs, n-MoSe_2_/p-WSe_2_, has exhibited the clear rectification with an ideality factor of ~2 at 290 K [246]. Similarly, p–n junction of p-WSe_2_/n-MoS_2_ (Fig. 7b) has also shown the promising gate tunable rectifying electrical characteristics [247–250]. The average conductance slope of 75 mV per decade at room temperature [250] and ideality factor of 1.2 were observed [251]. However, in recent work, the p–n heterojunction formed by *p-*type single-walled carbon nanotubes and *n-*type monolayer MoS_2_ has also revealed the charge transport behavior with a forward/reverse current ratio of larger than 10^4^ [252]. In addition, heterostructure made from InAs (*n* type)/WSe_2_ (*p* type) has displayed the even better rectification with a forward/reverse current ratio larger than 10^6^ and an ideality factor of ~1.1 [253].

**Fig. 8:**
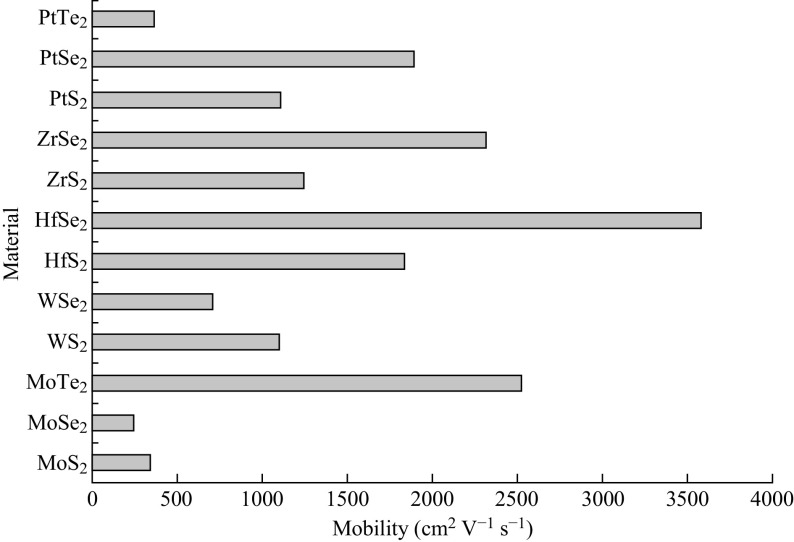
Theoretical charge carrier mobility of monolayer 2D TMDC materials. Redrawn from Ref. [254]

Secondly, to search and fabricate new materials may be another possible methodology to achieve high carrier mobility 2D materials. Recently, 2D material, InSe (metal chalcogenide)-based heterostructure encapsulated device has been reported, which demonstrates charge carrier mobility of ~10,000 and ~1000 cm^2^ V^−1^ s^−1^ at ~ 50 and 300 K, respectively [242]. Theoretical calculations have revealed that some new promising single-layer 2D TMDC materials may achieve higher mobility than that of MoS_2_ [254] as shown in Fig. 8. Certainly, the synthesis of these high-quality 2D materials is a challenge.

## References

[1] Wang QH, Kalantar-Zadeh K, Kis A, Coleman JN, Strano MS (2012). Electronics and optoelectronics of two-dimensional transition metal dichalcogenides. Nat. Nanotechnol..

[2] Wilson JA, Di Salvo FJ, Mahajan S (1974). Charge-density waves in metallic, layered, transition-metal dichalcogenides. Phys. Rev. Lett..

[3] Gupta A, Sakthivel T, Seal S (2015). Recent development in 2D materials beyond graphene. Prog. Mater Sci..

[4] Li X, Zhu H (2015). Two-dimensional MoS_2_: properties, preparation, and applications. J. Materiomics.

[5] Bhimanapati GR, Lin Z, Meunier V, Jung Y, Cha J (2015). Recent advances in two-dimensional materials beyond graphene. ACS Nano.

[6] Kim SJ, Choi K, Lee B, Kim Y, Hong BH (2015). Materials for flexible, stretchable electronics: graphene and 2D material. Annu. Rev. Mater. Res..

[7] Das S, Robinson JA, Dubey M, Terrones H, Terrones M (2015). Beyond Graphene: progress in novel two-dimensional materials and van der Waals solids. Annu. Rev. Mater. Res..

[8] Tong X, Ashalley E, Lin F, Li H, Wang ZM (2015). Advances in MoS_2_-based field effect transistors (FETs). Nano-Micro Lett..

[9] Butler SZ, Hollen SM, Cao L, Cui Y, Gupta JA (2013). Progress, challenges, and opportunities in two-dimensional materials beyond graphene. ACS Nano.

[10] Jariwala D, Sangwan VK, Lauhon LJ, Marks TJ, Hersam MC (2014). Emerging device applications for semiconducting two-dimensional transition metal dichalcogenides. ACS Nano.

[11] Xia F, Wang H, Xiao D, Dubey M, Ramasubramaniam A (2014). Two-dimensional material nanophotonics. Nat. Photon..

[12] Wang XR, Shi Y, Zhang R (2013). Field-effect transistors based on two-dimensional materials for logic applications. Chin. Phys. B.

[13] Fiori G, Bonaccorso F, Iannaccone G, Palacios T, Neumaier D, Seabaugh A, Banerjee SK, Colombo L (2014). Electronics based on two-dimensional materials. Nat. Nanotechnol..

[14] Nitin C, Muhammad RI, Narae K, Laurene T, Yeonwoong J, Saiful IK (2016). Two-dimensional lateral heterojunction through bandgap engineering of MoS_2_ via oxygen plasma. J. Phys.: Condens. Matter..

[15] Chang H-Y, Yogeesh MN, Ghosh R, Rai A, Sanne A, Yang S, Lu N, Banerjee SK, Akinwande D (2016). Large-area monolayer MoS_2_ for flexible low-power RF nanoelectronics in the GHz regime. Adv. Mater..

[16] Bonaccorso F, Colombo L, Yu G, Stoller M, Tozzini V, Ferrari AC, Ruoff RS, Pellegrini V (2015). Graphene, related two-dimensional crystals, and hybrid systems for energy conversion and storage. Science.

[17] Choudhary N, Patel MD, Park J, Sirota B, Choi W (2016). Synthesis of large scale MoS_2_ for electronics and energy applications. J. Mater. Res..

[18] Babu VJ, Vempati S, Uyar T, Ramakrishna S (2015). Review of one-dimensional and two-dimensional nanostructured materials for hydrogen generation. Phys. Chem. Chem. Phys..

[19] Varghese SS, Varghese SH, Swaminathan S, Singh KK, Mittal V (2015). Two-dimensional materials for sensing: graphene and beyond. Electronics.

[20] Xu M, Liang T, Shi M, Chen H (2013). Graphene-like two-dimensional materials. Chem. Rev..

[21] Bolotin KI, Sikes KJ, Jiang Z, Klima M, Fudenberg G, Hone J, Kim P, Stormer HL (2008). Ultrahigh electron mobility in suspended graphene. Solid State Commun..

[22] Wang X, Ouyang Y, Li X, Wang H, Guo J, Dai H (2008). Room-temperature all-semiconducting sub-10-nm graphene nanoribbon field-effect transistors. Phys. Rev. Lett..

[23] Ding Y, Wang Y, Ni J, Shi L, Shi S, Tang W (2011). First principles study of structural, vibrational and electronic properties of graphene-like MX_2_ (M = Mo, Nb, W, Ta; X = S, Se, Te) monolayers. Phys. B Condens. Matter..

[24] Ataca C, Şahin H, Ciraci S (2012). Stable, single-layer MX_2_ transition-metal oxides and dichalcogenides in a honeycomb-like structure. J. Phys. Chem. C.

[25] Splendiani A, Sun L, Zhang Y, Li T, Kim J, Chim C-Y, Galli G, Wang F (2010). Emerging photoluminescence in monolayer MoS_2_. Nano Lett..

[26] Mak KF, Lee C, Hone J, Shan J, Heinz TF (2010). Atomically thin MoS_2_: a new direct-gap semiconductor. Phys. Rev. Lett..

[27] Klots AR, Newaz AKM, Wang B, Prasai D, Krzyzanowska H (2014). Probing excitonic states in suspended two-dimensional semiconductors by photocurrent spectroscopy. Sci. Rep..

[28] Rasmussen FA, Thygesen KS (2015). Computational 2D materials database: electronic structure of transition-metal dichalcogenides and oxides. J. Phys. Chem. C.

[29] Novoselov KS, Jiang D, Schedin F, Booth TJ, Khotkevich VV, Morozov SV, Geim AK (2005). Two-dimensional atomic crystals. Proc. Natl. Acad. Sci..

[30] Radisavljevic B, Radenovic A, Brivio J, Giacometti V, Kis A (2011). Single-layer MoS_2_ transistors. Nat. Nanotechnol..

[31] Fuhrer MS, Hone J (2013). Measurement of mobility in dual-gated MoS_2_ transistors. Nat. Nanotechnol..

[32] Radisavljevic B, Kis A (2013). Reply to measurement of mobility in dual-gated MoS_2_ transistors. Nat. Nanotechnol..

[33] Yu Z, Ong Z-Y, Pan Y, Cui Y, Xin R (2016). Realization of room-temperature phonon-limited carrier transport in monolayer MoS_2_ by dielectric and carrier screening. Adv. Mater..

[34] Li X, Mullen JT, Jin Z, Borysenko KM, Nardelli MB, Kim KW (2013). Intrinsic electrical transport properties of monolayer silicene and MoS_2_ from first principles. Phys. Rev. B.

[35] Qiao J, Kong X, Hu Z-X, Yang F, Ji W (2014). High-mobility transport anisotropy and linear dichroism in few-layer black phosphorus. Nat. Commun..

[36] Li L, Yu Y, Ye GJ, Ge Q, Ou X, Wu H, Feng D, Chen XH, Zhang Y (2014). Black phosphorus field-effect transistors. Nat. Nanotechnol..

[37] Zhu W, Park S, Yogeesh MN, McNicholas KM, Bank SR, Akinwande D (2016). Black phosphorus flexible thin film transistors at gighertz frequencies. Nano Lett..

[38] Davis ME, Zuckerman JE, Choi CHJ, Seligson D, Tolcher A, Alabi CA, Yen Y, Heidel JD, Ribas A (2010). Evidence of RNAi in humans from systemically administered siRNA via targeted nanoparticles. Nature.

[39] Zou J, Liu J, Karakoti AS, Kumar A, Joung D, Li Q, Khondaker SI, Seal S, Zhai L (2010). Ultralight multiwalled carbon nanotube aerogel. ACS Nano.

[40] Karakoti AS, Tsigkou O, Yue S, Lee PD, Stevens MM, Jones JR, Seal S (2010). Rare earth oxides as nanoadditives in 3-D nanocomposite scaffolds for bone regeneration. J. Mater. Chem..

[41] Tiwari JN, Tiwari RN, Kim KS (2012). Zero-dimensional, one-dimensional, two-dimensional and three-dimensional nanostructured materials for advanced electrochemical energy devices. Prog. Mater. Sci..

[42] Mas Balleste R, Gomez Navarro C, Gomez Herrero J, Zamora F (2011). 2D materials: to graphene and beyond. Nanoscale.

[43] Novoselov KS, Geim AK, Morozov SV, Jiang D, Katsnelson MI, Grigorieva IV, Dubonos SV, Firsov AA (2005). Two-dimensional gas of massless Dirac fermions in graphene. Nature.

[44] Canadell E, LeBeuze A, El Khalifa MA, Chevrel R, Whangbo MH (1989). Origin of metal clustering in transition-metal chalcogenide layers MX_2_ (M = Nb, Ta, Mo, Re; X = S, Se). J. Am. Chem. Soc..

[45] Lukowski MA, Daniel AS, English CR, Meng F, Forticaux A, Hamers RJ, Jin S (2014). Highly active hydrogen evolution catalysis from metallic WS_2_ nanosheets. Energy Environ. Sci..

[46] Woodward RI, Howe RCT, Runcorn TH, Hu G, Torrisi F, Kelleher EJR, Hasan T (2015). Wideband saturable absorption in few-layer molybdenum diselenide (MoSe_2_) for Q-switching Yb-, Er- and Tm-doped fiber lasers. Opt. Express.

[47] Wilson J, Yoffe A (1969). The transition metal dichalcogenides discussion and interpretation of the observed optical, electrical and structural properties. Adv. Phys..

[48] Singh E, Kim KS, Yeom GY, Nalwa HS (2017). Two-dimensional transition metal dichalcogenide-based counter electrodes for dye-sensitized solar cells. RSC Adv..

[49] May P, Khan U, Coleman JN (2013). Reinforcement of metal with liquid-exfoliated inorganic nano-platelets. Appl. Phys. Lett..

[50] Choi W, Choudhary N, Han GH, Park J, Akinwande D, Lee YH (2017). Recent development of two-dimensional transition metal dichalcogenides and their applications. Mater. Today.

[51] Kappera R, Voiry D, Yalcin SE, Branch B, Gupta G, Mohite AD, Chhowalla M (2014). Phase-engineered low-resistance contacts for ultrathin MoS_2_ transistors. Nat. Mater..

[52] Yoon A, Lee Z (2017). Synthesis and properties of two dimensional doped transition metal dichalcogenides. Appl. Microsc..

[53] Kappera R, Voiry D, Yalcin SE, Jen W, Acerce M (2014). Metallic 1T phase source/drain electrodes for field effect transistors from chemical vapor deposited MoS_2_. APL Mater..

[54] Frey GL, Elani S, Homyonfer M, Feldman Y, Tenne R (1998). Optical-absorption spectra of inorganic fullerenelike MS_2_ (M = Mo, W). Phys. Rev. B.

[55] Ji F, Ren X, Zheng X, Liu Y, Pang L, Jiang J, Liu SF (2016). 2D-MoO_3_ nanosheets for superior gas sensors. Nanoscale.

[56] Wang J, Liu CJ (2015). Preparation of 2D WO_3_ Nanomaterials with enhanced catalytic activities: current status and perspective. ChemBioEng Rev..

[57] Choi S-J, Jang J-S, Park HJ, Kim I-D (2017). Optically sintered 2D RuO_2_ nanosheets: temperature-controlled NO_2_ reaction. Adv. Funct. Mater..

[58] Zhang Y, Wu W, Zhang K, Liu C, Yu A, Peng M, Zhai J (2016). Raman study of 2D anatase TiO_2_ nanosheets. Phys. Chem. Chem. Phys..

[59] Liu Z, Xu K, She P, Yin S, Zhu X, Sun H (2016). Self-assembly of 2D MnO_2_ nanosheets into high-purity aerogels with ultralow density. Chem. Sci..

[60] Yang G, Song H, Wu M, Wang C (2016). SnO_2_ nanoparticles anchored on 2D V_2_O_5_ nanosheets with enhanced lithium-storage performances. Electrochim. Acta.

[61] Xu X, Takada K, Fukuda K, Ohnishi T, Akatsuka K, Osada M, Hang BT, Kumagai K, Sekiguchi T, Sasaki T (2011). Tantalum oxide nanomesh as self-standing one nanometre thick electrolyte. Energy Environ. Sci..

[62] Eda G, Fujita T, Yamaguchi H, Voiry D, Chen M, Chhowalla M (2012). Coherent atomic and electronic heterostructures of single-layer MoS_2_. ACS Nano.

[63] Li H, Lu G, Yin Z, He Q, Li H, Zhang Q, Zhang H (2012). Optical identification of single- and few-layer MoS_2_ sheets. Small.

[64] Tongay S, Zhou J, Ataca C, Lo K, Matthews TS, Li J, Grossman JC, Wu J (2012). Thermally driven crossover from indirect toward direct bandgap in 2D semiconductors: MoSe_2_ versus MoS_2_. Nano Lett..

[65] Mak KF, Shan J (2016). Photonics and optoelectronics of 2D semiconductor transition metal dichalcogenides. Nat. Photon..

[66] Osada M, Sasaki T (2012). Two-dimensional dielectric nanosheets: novel nanoelectronics from nanocrystal building blocks. Adv. Mater..

[67] Gordon RA, Yang D, Crozier ED, Jiang DT, Frindt RF (2002). Structures of exfoliated single layers of WS_2_, MoS_2_, and MoSe_2_ in aqueous suspension. Phys. Rev. B.

[68] Gamble FR, Osiecki JH, Cais M, Pisharody R, DiSalvo FJ, Geballe TH (1971). Intercalation complexes of lewis bases and layered sulfides: a large class of new superconductors. Science.

[69] Keum DH, Cho S, Kim JH, Choe D-H, Sung H-J (2015). Bandgap opening in few-layered monoclinic MoTe_2_. Nat. Phys..

[70] Empante TA, Zhou Y, Klee V, Nguyen AE, Lu IH (2017). Chemical vapor deposition growth of few-layer MoTe_2_ in the 2H, 1T′, and 1T phases: tunable properties of MoTe_2_ films. ACS Nano.

[71] Enyashin AN, Yadgarov L, Houben L, Popov I, Weidenbach M, Tenne R, Bar-Sadan M, Seifert G (2011). New route for stabilization of 1T-WS_2_ and MoS_2_ phases. J. Phys. Chem. C.

[72] Kong D, Dang W, Cha JJ, Li H, Meister S, Peng H, Liu Z, Cui Y (2010). Few-layer nanoplates of Bi_2_Se_3_ and Bi_2_Te_3_ with highly tunable chemical potential. Nano Lett..

[73] Yang S, Gong Y, Liu Z, Zhan L, Hashim DP, Ma L, Vajtai R, Ajayan PM (2013). Bottom-up approach toward single-crystalline VO_2_-graphene ribbons as cathodes for ultrafast lithium storage. Nano Lett..

[74] Taha-Tijerina J, Narayanan TN, Gao G, Rohde M, Tsentalovich DA, Pasquali M, Ajayan PM (2012). Electrically insulating thermal nano-oils using 2D fillers. ACS Nano.

[75] Ebina Y, Sasaki T, Harada M, Watanabe M (2002). Restacked perovskite nanosheets and their Pt-loaded materials as photocatalysts. Chem. Mater..

[76] Ozawa TC, Fukuda K, Akatsuka K, Ebina Y, Sasaki T (2007). Preparation and characterization of the Eu^3+^ doped perovskite nanosheet phosphor: La_0.90_Eu_0.05_Nb_2_O_7_. Chem. Mater..

[77] Hsu W-T, Zhao Z-A, Li L-J, Chen C-H, Chiu M-H, Chang P-S, Chou Y-C, Chang W-H (2014). Second harmonic generation from artificially stacked transition metal dichalcogenide twisted bilayers. ACS Nano.

[78] Acun A, Poelsema B, Zandvliet HJW, van Gastel R (2013). The instability of silicene on Ag (111). Appl. Phys. Lett..

[79] Tao L, Cinquanta E, Chiappe D, Grazianetti C, Fanciulli M, Dubey M, Molle A, Akinwande D (2015). Silicene field-effect transistors operating at room temperature. Nat. Nanotechnol..

[80] Oughaddou H, Aufray B, Bibérian JP, Hoarau JY (1999). Growth mode and dissolution kinetics of germanium thin films on Ag(001) surface: an AES–LEED investigation. Surf. Sci..

[81] Golias E, Xenogiannopoulou E, Tsoutsou D, Tsipas P, Giamini SA, Dimoulas A (2013). Surface electronic bands of submonolayer Ge on Ag(111). Phys. Rev. B.

[82] Hao B, Yan Y, Wang X, Chen G (2013). Synthesis of anatase TiO_2_ nanosheets with enhanced pseudocapacitive contribution for fast Lithium storage. ACS Appl. Mater. Interfaces..

[83] Sterrer M, Risse T, Martinez Pozzoni U, Giordano L, Heyde M, Rust H-P, Pacchioni G, Freund H-J (2007). Control of the charge state of metal atoms on thin MgO films. Phys. Rev. Lett..

[84] Jung H, Park J, Oh I-K, Choi T, Lee S, Hong J, Lee T, Kim S-H, Kim H (2014). Fabrication of transferable Al_2_O_3_ nanosheet by atomic layer deposition for graphene FET. ACS Appl. Mater. Interfaces.

[85] Novoselov KS, Geim AK, Morozov SV, Jiang D, Zhang Y, Dubonos SV, Grigorieva IV, Firsov AA (2004). Electric field effect in atomically thin carbon films. Science.

[86] Miremadi BK, Morrison SR (1987). High activity catalyst from exfoliated MoS_2_. J. Catalysis.

[87] Late DJ (2014). Temperature dependent phonon shifts in single-layer WS_2_. ACS Appl. Mater. Interfaces..

[88] De Padova P, Ottaviani C, Quaresima C, Olivieri B, Imperatori P (2014). 24 h stability of thick multilayer silicene in air. 2D Mater..

[89] Coleman JN, Lotya M, Neill AO, Bergin SD, King PJ (2011). Two-dimensional nanosheets produced by liquid exfoliation of layered materials. Science.

[90] Zhou KG, Mao N-N, Wang H-X, Peng Y, Zhang H-L (2011). A mixed-solvent strategy for efficient exfoliation of inorganic graphene analogues. Angew. Chem. Int. Ed..

[91] Smith RJ, King PJ, Lotya M, Wirtz C, Khan U (2011). Large-scale exfoliation of inorganic layered compounds in aqueous surfactant solutions. Adv. Mater..

[92] Brodie BC (1859). On the atomic weight of graphite. Philos. Trans. R. Soc. London.

[93] Luan VH, Tien HN, Hoa LT, Hien NTM, Oh E-S (2013). Synthesis of a highly conductive and large surface area graphene oxide hydrogel and its use in a supercapacitor. J. Mater. Chem. A.

[94] Hummers WS, Offeman RE (1958). Preparation of graphitic oxide. J. Am. Chem. Soc..

[95] Goncalves G, Marques PAAP, Granadeiro CM, Nogueira HIS, Singh MK, Grácio J (2009). Surface modification of graphene nanosheets with gold nanoparticles: the role of oxygen moieties at graphene surface on gold nucleation and growth. Chem. Mater..

[96] Xiong F, Wang H, Liu X, Sun J, Brongersma M, Pop E, Cui Y (2015). Li Intercalation in MoS_2_: in Situ observation of its dynamics and tuning optical and electrical properties. Nano Lett..

[97] Petrović M, Šrut Rakić I, Runte S, Busse C, Sadowski JT (2013). The mechanism of caesium intercalation of graphene. Nat. Commun..

[98] Nicolosi V, Chhowalla M, Kanatzidis MG, Strano MS, Coleman JN (2013). Liquid exfoliation of layered materials. Science.

[99] Zhan Y, Liu Z, Najmaei S, Ajayan PM, Lou J (2012). Large-area vapor-phase growth and characterization of MoS_2_ atomic layers on a SiO_2_ substrate. Small.

[100] Lee YH, Zhang XQ, Zhang W, Chang MT, Lin CT (2012). Synthesis of large-area MoS_2_ atomic layers with chemical vapor deposition. Adv. Mater..

[101] Liu KK, Zhang W, Lee YH, Lin YC, Chang MT (2012). Growth of large-area and highly crystalline MoS_2_ thin layers on insulating substrates. Nano Lett..

[102] Peters ES, Carmalt CJ, Parkin IP (2004). Dual-source chemical vapour deposition of titanium sulfide thin films from tetrakisdimethylamidotitanium and sulfur precursors. J. Mater. Chem..

[103] Boscher ND, Blackman CS, Carmalt CJ, Parkin IP, Prieto AG (2007). Atmospheric pressure chemical vapour deposition of vanadium diselenide thin films. Appl. Surf. Sci..

[104] Boscher ND, Carmalt CJ, Parkin IP (2006). Atmospheric pressure chemical vapor deposition of WSe_2_ thin films on glass-highly hydrophobic sticky surfaces. J. Mater. Chem..

[105] Carmalt CJ, Parkin IP, Peters ES (2003). Atmospheric pressure chemical vapour deposition of WS_2_ thin films on glass. Polyhedron.

[106] Boscher ND, Carmalt CJ, Palgrave RG, Gil-Tomas JJ, Parkin IP (2006). Atmospheric pressure CVD of molybdenum diselenide films on glass. Chem. Vapor Depos..

[107] Müller F, Stöwe K, Sachdev H (2005). Symmetry versus commensurability: epitaxial growth of hexagonal boron nitride on Pt (111) from B-trichloroborazine (ClBNH)3. Chem. Mater..

[108] Auwärter W, Suter HU, Sachdev H, Greber T (2004). Synthesis of one monolayer of hexagonal boron nitride on Ni (111) from B-trichloroborazine (ClBNH)_3_. Chem. Mater..

[109] Nagashima A, Tejima N, Gamou Y, Kawai T, Oshima C (1996). Electronic states of monolayer hexagonal boron nitride formed on the metal surfaces. Surf. Sci..

[110] Shi Y, Zhou W, Lu A-Y, Fang W, Lee Y-H (2012). Van der Waals epitaxy of MoS_2_ layers using graphene as growth templates. Nano Lett..

[111] Zheng Y, Koëbel A, Pétroff JF, Boulliard JC, Capelle B, Eddrief M (1996). GaSeSi (111) heteroepitaxy: the early stages of growth. J. Cryst. Growth.

[112] Lalmi B, Oughaddou H, Enriquez H, Kara A, Vizzini S, Ealet B, Aufray B (2010). Epitaxial growth of a silicene sheet. Appl. Phys. Lett..

[113] Léandri C, Oughaddou H, Aufray B, Gay JM, Le Lay G, Ranguis A, Garreau Y (2007). Growth of Si nanostructures on Ag(001). Surf. Sci..

[114] Aufray B, Kara A, Vizzini S, Oughaddou H, Léandri C, Ealet B, Le Lay G (2010). Graphene-like silicon nanoribbons on Ag(110): a possible formation of silicene. Appl. Phys. Lett..

[115] De Padova P, Kubo O, Olivieri B, Quaresima C, Nakayama T, Aono M, Le Lay G (2012). Multilayer silicene nanoribbons. Nano Lett..

[116] Oughaddou H, Gay JM, Aufray B, Lapena L, Le Lay G, Bunk O, Falkenberg G, Zeysing JH, Johnson RL (2000). Ge tetramer structure of the p(2√2 × 4√2)R45° surface reconstruction of Ge/Ag(001): a surface x-ray diffraction and STM study. Phys. Rev. B.

[117] Peng Y, Meng Z, Zhong C, Lu J, Yu W, Jia Y, Qian Y (2001). Hydrothermal synthesis and characterization of single-molecular-layer MoS_2_ and MoSe_2_. Chem. Lett..

[118] Li Y, Wang H, Xie L, Liang Y, Hong G, Dai H (2011). MoS_2_ nanoparticles grown on graphene: an advanced catalyst for the hydrogen evolution reaction. J. Am. Chem. Soc..

[119] Jeong S, Yoo D, Jang J-T, Kim M, Cheon J (2012). Well-defined colloidal 2-D layered transition-metal chalcogenide nanocrystals via generalized synthetic protocols. J. Am. Chem. Soc..

[120] Qiliang L, Sang-Mo K, Richter CA, Edelstein MD, Bonevich JE, Kopanski JJ, Suehle JS, Vogel EM (2007). Precise alignment of single nanowires and fabrication of nanoelectromechanical switch and other test structures. IEEE Trans. Nanotechnol..

[121] Jun Y-W, Seo J-W, Oh SJ, Cheon J (2005). Recent advances in the shape control of inorganic nano-building blocks. Coordin. Chem. Rev..

[122] Xu Y, Liu Z, Zhang X, Wang Y, Tian J, Huang Y, Ma Y, Zhang X, Chen Y (2009). A graphene hybrid material covalently functionalized with porphyrin: synthesis and optical limiting property. Adv. Mater..

[123] Hernandez Y, Nicolosi V, Lotya M, Blighe FM, Sun Z (2008). High-yield production of graphene by liquid-phase exfoliation of graphite. Nat. Nanotechnol..

[124] Dikin DA, Stankovich S, Zimney EJ, Piner RD, Dommett GHB, Evmenenko G, Nguyen ST, Ruoff RS (2007). Preparation and characterization of graphene oxide paper. Nature.

[125] Xiang Q, Yu J, Jaroniec M (2012). Synergetic effect of MoS_2_ and graphene as cocatalysts for enhanced photocatalytic H_2_ production activity of TiO_2_ nanoparticles. J. Am. Chem. Soc..

[126] Wang G, Shen X, Yao J, Park J (2009). Graphene nanosheets for enhanced lithium storage in lithium ion batteries. Carbon.

[127] Zhou G, Li F, Cheng H-M (2014). Progress in flexible lithium batteries and future prospects. Energy Environ. Sci..

[128] Patel MD, Cha E, Choudhary N, Kang C, Lee W, Hwang JY, Choi W (2016). Vertically oriented MoS_2_ nanoflakes coated on 3D carbon nanotubes for next generation Li-ion batteries. Nanotechnology.

[129] Yoo JJ, Balakrishnan K, Huang J, Meunier V, Sumpter BG (2011). Ultrathin planar graphene supercapacitors. Nano Lett..

[130] Mhamdi A, Salem EB, Jaziri S (2013). Electronic reflection for a single-layer graphene quantum well. Solid State Commun..

[131] Geim AK (2009). Graphene: status and prospects. Science.

[132] Zhang Y, Tang T-T, Girit C, Hao Z, Martin MC, Zettl A, Crommie MF, Shen YR, Wang F (2009). Direct observation of a widely tunable bandgap in bilayer graphene. Nature.

[133] Mohiuddin TMG, Lombardo A, Nair RR, Bonetti A, Savini G (2009). Uniaxial strain in graphene by raman spectroscopy: G peak splitting, gruneisen parameters, and sample orientation. Phys. Rev. B.

[134] Conley HJ, Wang B, Ziegler JI, Haglund RF, Pantelides ST, Bolotin KI (2013). Bandgap engineering of strained monolayer and bilayer MoS_2_. Nano Lett..

[135] Li X, Wang X, Zhang L, Lee S, Dai H (2008). Chemically derived, ultrasmooth graphene nanoribbon semiconductors. Science.

[136] Akis R, Ferry DK (2008). Using magnetic fields and band gap engineering to achieve robust spin filtering in finite quantum dot arrays. J. Phys: Conf. Ser..

[137] Meyer JC, Geim AK, Katsnelson MI, Novoselov KS, Booth TJ, Roth S (2007). The structure of suspended graphene sheets. Nature.

[138] Du X, Skachko I, Barker A, Andrei EY (2008). Approaching ballistic transport in suspended graphene. Nat. Nanotechnol..

[139] Kam KK, Parkinson BA (1982). Detailed photocurrent spectroscopy of the semiconducting group VIB transition metal dichalcogenides. J. Phys. Chem..

[140] K. Uchida, H. Watanabe, A. Kinoshita, J. Koga, T. Numata, S. Takagi, Experimental study on carrier transport mechanism in ultrathin-body SOI nand p-MOSFETs with SOI thickness less than 5 nm. IEEE Int. Electron Devices Meeting (IEDM), pp. 47–50 (2002). doi:10.1109/IEDM.2002.1175776

[141] Yin D, Yoon Y (2016). Design strategy of two-dimensional material field-effect transistors: engineering the number of layers in phosphorene FETs. J. Appl. Phys..

[142] Chung TF, Shen T, Cao H, Luis A, Wu W, Yu Q, Newell D, Chen YP (2013). Synthetic graphene grown by chemical vapor deposition on copper foils. Inter. J. Mod. Phys. B.

[143] Kalantar-Zadeh K, Ou JZ (2016). Biosensors based on two-dimensional MoS_2_. ACS Sens..

[144] Reina A, Jia X, Ho J, Nezich D, Son H, Bulovic V, Dresselhaus MS, Kong J (2009). Large area, few-layer graphene films on arbitrary substrates by chemical vapor deposition. Nano Lett..

[145] Mao N, Chen Y, Liu D, Zhang J, Xie L (2013). Solvatochromic effect on the photoluminescence of MoS_2_ monolayers. Small.

[146] Zhang H, Bao Q, Tang D, Zhao L, Loh K (2009). Large energy soliton erbium-doped fiber laser with a graphene-polymer composite mode locker. Appl. Phys. Lett..

[147] Lu S, Zhao C, Zou Y, Chen S, Chen Y, Li Y, Zhang H, Wen S, Tang D (2013). Third order nonlinear optical property of Bi_2_Se_3_. Opt. Express.

[148] Zhang H, Lu SB, Zheng J, Du J, Wen SC, Tang DY, Loh KP (2014). Molybdenum disulfide (MoS_2_) as a broadband saturable absorber for ultra-fast photonics. Opt. Express.

[149] Lu SB, Miao LL, Guo ZN, Qi X, Zhao CJ, Zhang H, Wen SC, Tang DY, Fan DY (2015). Broadband nonlinear optical response in multi-layer black phosphorus: an emerging infrared and mid-infrared optical material. Opt. Express.

[150] Nair RR, Blake P, Grigorenko AN, Novoselov KS, Booth TJ, Stauber T, Peres NMR, Geim AK (2008). Fine structure constant defines visual transparency of graphene. Science.

[151] Eda G, Fanchini G, Chhowalla M (2008). Large-area ultrathin films of reduced graphene oxide as a transparent and flexible electronic material. Nat. Nanotechnol..

[152] Lee C, Wei X, Kysar JW, Hone J (2008). Measurement of the elastic properties and intrinsic strength of monolayer graphene. Science.

[153] Gómez-Navarro C, Burghard M, Kern K (2008). Elastic properties of chemically derived single graphene sheets. Nano Lett..

[154] An Introduction to FETs. Radio Commun. **76**(7), 1–5 (2000). http://www.colorado.edu/physics/phys3330/phys3330_sp15/resources/AN101FETintro.pdf (accessed)

[155] Ferain I, Colinge CA, Colinge J-P (2011). Multigate transistors as the future of classical metal-oxide-semiconductor field-effect transistors. Nature.

[156] Wilk GD, Wallace RM, Anthony JM (2001). High-κ gate dielectrics: current status and materials properties considerations. J. Appl. Phys..

[157] Seabaugh AC, Zhang Q (2010). Low-voltage tunnel transistors for beyond CMOS logic. Proc. IEEE.

[158] Ionescu AM, Riel H (2011). Tunnel field-effect transistors as energy-efficient electronic switches. Nature.

[159] Ghosh RK, Mahapatra S (2013). Monolayer transition metal dichalcogenide channel-based tunnel transistor. IEEE J. Electron Devices Soc..

[160] Zhang Q, Iannaccone G, Fiori G (2014). Two-dimensional tunnel transistors based on Bi_2_Se_3_ thin film. IEEE Electron Device Lett..

[161] Song WG, Kwon H-J, Park J, Yeo J, Kim M (2016). High-performance flexible multilayer MoS_2_ transistors on solution-based polyimide substrates. Adv. Funct. Mater..

[162] Hong YK, Yoo G, Kwon J, Hong S, Song WG (2016). High performance and transparent multilayer MoS_2_ transistors: tuning Schottky barrier characteristics. AIP Adv..

[163] Liu L, Lu Y, Guo J (2013). On monolayer MoS_2_ field-effect transistors at the scaling limit. IEEE Trans. Electron Devices.

[164] L. Kai-Shin, W. Bo-Wei, L. Lain-Jong, L. Ming-Yang, C. Chia-Chin Kevin, et al., MoS_2_ U-shape MOSFET with 10 nm channel length and poly-Si source/drain serving as seed for full wafer CVD MoS_2_ availability. In Proc. IEEE Symp. VLSI Technol., pp. 1–2 (2016). doi:10.1109/VLSIT.2016.7573375

[165] Novoselov KS, Falko VI, Colombo L, Gellert PR, Schwab MG, Kim K (2012). A roadmap for graphene. Nature.

[166] Liu L, Kumar SB, Ouyang Y, Guo J (2011). Performance limits of monolayer transition metal dichalcogenide transistors. IEEE Trans. Electron Devices.

[167] Naveh Y, Likharev K (2000). Modeling of 10-nm-scale ballistic MOSFET’s. IEEE Electron Device Lett..

[168] Di Bartolomeo A (2016). Graphene schottky diodes: an experimental review of the rectifying graphene/semiconductor heterojunction. Phys. Rep..

[169] Balandin AA, Ghosh S, Bao W, Calizo I, Teweldebrhan D, Miao F, Lau CN (2008). Superior thermal conductivity of single-layer graphene. Nano Lett..

[170] Jo I, Pettes MT, Kim J, Watanabe K, Taniguchi T, Yao Z, Shi L (2013). Thermal conductivity and phonon transport in suspended few-layer hexagonal boron nitride. Nano Lett..

[171] Ghosh S, Calizo I, Teweldebrhan D, Pokatilov EP, Nika DL, Balandin AA, Bao W, Miao F, Lau CN (2008). Extremely high thermal conductivity of graphene: prospects for thermal management applications in nanoelectronic circuits. Appl. Phys. Lett..

[172] Lan J, Wang J-S, Gan CK, Chin SK (2009). Edge effects on quantum thermal transport in graphene nanoribbons: tight-binding calculations. Phys. Rev. B.

[173] Nika DL, Pokatilov EP, Askerov AS, Balandin AA (2009). Phonon thermal conduction in graphene: role of umklapp and edge roughness scattering. Phys. Rev. B.

[174] Cai Y, Lan J, Zhang G, Zhang Y-W (2014). Lattice vibrational modes and phonon thermal conductivity of monolayer MoS_2_. Phys. Rev. B.

[175] Liu X, Zhang G, Pei Q-X, Zhang Y-W (2013). Phonon thermal conductivity of monolayer MoS_2_ sheet and nanoribbons. Appl. Phys. Lett..

[176] Jiang J-W, Park HS, Rabczuk T (2013). Molecular dynamics simulations of single-layer molybdenum disulphide (MoS_2_): stillinger-weber parametrization, mechanical properties, and thermal conductivity. J. Appl. Phys..

[177] Kim J-Y, Choi S-M, Seo W-S, Cho W-S (2010). Thermal and electronic properties of exfoliated metal chalcogenides. Bull. Korean Chem. Soc..

[178] Sahoo S, Gaur AP, Ahmadi M, Guinel MJ-F, Katiyar RS (2013). Temperature-dependent raman studies and thermal conductivity of few-layer MoS_2_. J. Phys. Chem. C.

[179] Zong Z, Li L, Jang J, Lu N, Liu M (2015). Analytical surface-potential compact model for amorphous-IGZO thin-film transistors. J. Appl. Phys..

[180] Krishnamoorthy S, Chowdhury MH (2009). Investigation and a practical compact network model of thermal stress in integrated circuits. Integr. Comput.-Aided Eng..

[181] Qian X, Wang Y, Li W, Lu J, Li J (2015). Modelling of stacked 2D materials and devices. 2D Mater..

[182] Lu N, Sun P, Li L, Liu Q, Long S, Hangbing L, Liu M (2016). Thermal effect on endurance performance of 3-dimensional RRAM crossbar array. Chin. Phys. B.

[183] Buccella P, Stefanucci C, Zou H, Moursy Y, Iskander R, Sallese J-M, Kayal M (2016). Methodology for 3-D substrate network extraction for spice simulation of parasitic currents in smart power ICs. IEEE Trans. Comput.-Aided Des. Integr. Circuits Syst..

[184] Nianduan L, Lingfei W, Ling L, Ming L (2017). A review for compact model of graphene field-effect transistors. Chin. Phys. B.

[185] Yoon Y, Ganapathi K, Salahuddin S (2011). How good can monolayer MoS_2_ transistors Be. Nano Lett..

[186] Ma N, Jena D (2015). Carrier statistics and quantum capacitance effects on mobility extraction in two-dimensional crystal semiconductor field-effect transistors. 2D Mater..

[187] Taur Y, Wu J, Min J (2016). A Short-Channel I-V Model for 2-D MOSFETs. IEEE Trans. Electron Devices.

[188] Jiménez D (2012). Drift-diffusion model for single layer transition metal dichalcogenide field-effect transistors. Appl. Phys. Lett..

[189] C. Kshirsagar, W. Xu, C. Kim, S. Koester, Design and analysis of MoS_2_-based MOSFETs for ultra-low-leakage dynamic memory applications. In 72nd Annu. Device Res. Conf., pp.187–188 (2014). doi:10.1109/DRC.2014.6872360

[190] Cao W, Kang J, Liu W, Banerjee K (2014). A compact current–voltage model for 2D semiconductor based field-effect transistors considering interface traps, mobility degradation, and inefficient doping effect. IEEE Trans. Electron Devices.

[191] Yadav C, Agarwal A, Chauhan YS (2017). Compact modeling of transition metal dichalcogenide based thin body transistors and circuit validation. IEEE Trans. Electron Devices.

[192] Schwierz F (2010). Graphene transistors. Nat. Nanotechnol..

[193] Ayari A, Cobas E, Ogundadegbe O, Fuhrer MS (2007). Realization and electrical characterization of ultrathin crystals of layered transition-metal dichalcogenides. J. Appl. Phys..

[194] Radisavljevic B, Whitwick MB, Kis A (2013). Correction to integrated circuits and logic operations based on single-layer MoS_2_. ACS Nano.

[195] Lopez-Sanchez O, Lembke D, Kayci M, Radenovic A, Kis A (2013). Ultrasensitive photodetectors based on monolayer MoS_2_. Nat. Nanotechnol..

[196] Yu WJ, Liu Y, Zhou H, Yin A, Li Z, Huang Y, Duan X (2013). Highly efficient gate-tunable photocurrent generation in vertical heterostructures of layered materials. Nat. Nanotechnol..

[197] Esmaeili-Rad MR, Salahuddin S (2013). High performance molybdenum disulfide amorphous silicon heterojunction photodetector. Sci. Rep..

[198] Liu B, Fathi M, Chen L, Abbas A, Ma Y, Zhou C (2015). Chemical vapor deposition growth of monolayer WSe_2_ with tunable device characteristics and growth mechanism study. ACS Nano.

[199] Fang H, Chuang S, Chang TC, Takei K, Takahashi T, Javey A (2012). High-performance single layered WSe_2_ p-FETs with chemically doped contacts. Nano Lett..

[200] Podzorov V, Gershenson M, Kloc C, Zeis R, Bucher E (2004). High-mobility field-effect transistors based on transition metal dichalcogenides. Appl. Phys. Lett..

[201] Huang J-K, Pu J, Hsu C-L, Chiu M-H, Juang Z-Y (2014). Large-area synthesis of highly crystalline WSe_2_ monolayers and device applications. ACS Nano.

[202] Bernède JC, Kettaf M, Khelil A, Spiesser M (1996). p-n junctions in molybdenum ditelluride. Phys. Status Solidi A.

[203] Conan A, Bonnet A, Zoaeter M, Ramoul D (1984). Dependence of the total mobility in a one-band model applicationto n-type MoTe_2_. Phys. Status Solidi B.

[204] Lin Y-F, Xu Y, Lin C-Y, Suen Y-W, Yamamoto M, Nakaharai S, Ueno K, Tsukagoshi K (2015). Origin of noise in layered MoTe_2_ transistors and its possible use for environmental sensors. Adv. Mater..

[205] Lin Y-F, Xu Y, Wang S-T, Li S-L, Yamamoto M (2014). Ambipolar MoTe_2_ transistors and their applications in logic circuits. Adv. Mater..

[206] Liu H, Neal AT, Ye PD (2012). Channel length scaling of MoS_2_ MOSFETs. ACS Nano.

[207] Ghatak S, Pal AN, Ghosh A (2011). Nature of electronic states in atomically thin MoS_2_ field-effect transistors. ACS Nano.

[208] Liu H, Ye PD (2012). MoS_2_ dual-gate MOSFET with atomic-layer-deposited Al_2_O_3_ as top-gate dielectric. IEEE Electron Device Lett..

[209] Lee K, Kim H-Y, Lotya M, Coleman JN, Kim G-T, Duesberg GS (2011). Electrical characteristics of molybdenum disulfide flakes produced by liquid exfoliation. Adv. Mater..

[210] Perera MM, Lin M-W, Chuang H-J, Chamlagain BP, Wang C, Tan X, Cheng MM-C, Tománek D, Zhou Z (2013). Improved carrier mobility in few-layer MoS_2_ field-effect transistors with ionic-liquid gating. ACS Nano.

[211] Lee G-H, Yu Y-J, Cui X, Petrone N, Lee C-H (2013). Flexible and transparent MoS_2_ field-effect transistors on hexagonal boron nitride-graphene heterostructures. ACS Nano.

[212] Li S-L, Wakabayashi K, Xu Y, Nakaharai S, Komatsu K, Li W-W, Lin Y-F, Aparecido-Ferreira A, Tsukagoshi K (2013). Thickness-dependent interfacial coulomb scattering in atomically thin field-effect transistors. Nano Lett..

[213] Li S-L, Tsukagoshi K (2015). Carrier injection and scattering in atomically thin chalcogenides. J. Phys. Soc. Jpn..

[214] Cui X, Lee G-H, Kim YD, Arefe G, Huang PY (2015). Multi-terminal transport measurements of MoS_2_ using a van der Waals heterostructure device platform. Nat. Nanotechnol..

[215] Kaasbjerg K, Thygesen KS, Jacobsen KW (2012). Phonon-limited mobility in n-type single-layer MoS_2_ from first principles. Phys. Rev. B.

[216] Baugher BWH, Churchill HOH, Yang Y, Jarillo-Herrero P (2013). Intrinsic electronic transport properties of high-quality monolayer and bilayer MoS_2_. Nano Lett..

[217] Larentis S, Fallahazad B, Tutuc E (2012). Field-effect transistors and intrinsic mobility in ultra-thin MoSe_2_ layers. Appl. Phys. Lett..

[218] Ye JT, Zhang YJ, Akashi R, Bahramy MS, Arita R, Iwasa Y (2012). Superconducting dome in a gate-tuned band insulator. Science.

[219] Radisavljevic B, Kis A (2013). Mobility engineering and a metal–insulator transition in monolayer MoS_2_. Nat. Mater..

[220] Li S-L, Komatsu K, Nakaharai S, Lin Y-F, Yamamoto M, Duan X, Tsukagoshi K (2014). Thickness scaling effect on interfacial barrier and electrical contact to two-dimensional MoS_2_ layers. ACS Nano.

[221] Sze SM, Ng KK (2007). Physics of Semiconductor Devices.

[222] Heinze S, Tersoff J, Martel R, Derycke V, Appenzeller J, Avouris P (2002). Carbon nanotubes as Schottky barrier transistors. Phys. Rev. Lett..

[223] Guo Y, Han Y, Li J, Xiang A, Wei X, Gao S, Chen Q (2014). Study on the resistance distribution at the contact between molybdenum disulfide and metals. ACS Nano.

[224] Dankert A, Langouche L, Kamalakar MV, Dash SP (2014). High-performance molybdenum disulfide field-effect transistors with spin tunnel contacts. ACS Nano.

[225] Chen J-R, Odenthal PM, Swartz AG, Floyd GC, Wen H, Luo KY, Kawakami RK (2013). Control of schottky barriers in single layer MoS_2_ transistors with ferromagnetic contacts. Nano Lett..

[226] Gold A (1987). Electronic transport properties of a two-dimensional electron gas in a silicon quantum-well structure at low temperature. Phys. Rev. B.

[227] Ando T, Fowler AB, Stern F (1982). Electronic properties of two-dimensional systems. Rev. Mod. Phys..

[228] Das Sarma S, Adam S, Hwang EH, Rossi E (2011). Electronic transport in two-dimensional graphene. Rev. Mod. Phys..

[229] Kaasbjerg K, Thygesen KS, Jauho A-P (2013). Acoustic phonon limited mobility in two-dimensional semiconductors: deformation potential and piezoelectric scattering in monolayer MoS_2_ from first principles. Phys. Rev. B.

[230] Ma N, Jena D (2014). Charge scattering and mobility in atomically thin semiconductors. Phys. Rev. X.

[231] DaSilva AM, Zou K, Jain JK, Zhu J (2010). Mechanism for current saturation and energy dissipation in graphene transistors. Phys. Rev. Lett..

[232] Moore BT, Ferry DK (1980). Remote polar phonon scattering in Si inversion layers. J. Appl. Phys..

[233] Hong J, Hu Z, Probert M, Li K, Lv D (2015). Exploring atomic defects in molybdenum disulphide monolayers. Nat. Commun..

[234] Schmidt H, Wang S, Chu L, Toh M, Kumar R (2014). Transport properties of monolayer MoS_2_ grown by chemical vapor deposition. Nano Lett..

[235] Hwang EH, Adam S, Sarma SD (2007). Carrier transport in two-dimensional graphene layers. Phys. Rev. Lett..

[236] Zou X, Liu Y, Yakobson BI (2013). Predicting dislocations and grain boundaries in two-dimensional metal-disulfides from the first principles. Nano Lett..

[237] Shi Y, Li H, Li L-J (2015). Recent advances in controlled synthesis of two-dimensional transition metal dichalcogenides via vapour deposition techniques. Chem. Soc. Rev..

[238] Yu J, Li J, Zhang W, Chang H (2015). Synthesis of high quality two-dimensional materials via chemical vapor deposition. Chem. Sci..

[239] Chen W, Zhao J, Zhang J, Gu L, Yang Z (2015). Oxygen-assisted chemical vapor deposition growth of large single-crystal and high-quality monolayer MoS_2_. J. Am. Chem. Soc..

[240] Bilgin I, Liu F, Vargas A, Winchester A, Man MKL (2015). Chemical vapor deposition synthesized atomically thin molybdenum disulfide with optoelectronic-grade crystalline quality. ACS Nano.

[241] Yu Y, Li C, Liu Y, Su L, Zhang Y, Cao L (2013). Controlled scalable synthesis of uniform, high-quality monolayer and few-layer MoS_2_ films. Sci. Rep..

[242] Bandurin DA, Tyurnina AV, Yu GL, Mishchenko A, Zólyomi V, Morozov SV (2017). High electron mobility, quantum hall effect and anomalous optical response in atomically thin InSe. Nat. Nanotechnol..

[243] Diaz HC, Addou R, Batzill M (2014). Interface properties of CVD grown graphene transferred onto MoS_2_ (0001). Nanoscale.

[244] Choudhary N, Park J, Hwang JY, Chung H-S, Dumas KH, Khondaker SI, Choi W, Jung Y (2016). Centimeter scale patterned growth of vertically stacked few layer only 2D MoS_2_/WS_2_ van der Waals heterostructure. Sci. Rep..

[245] Roy T, Tosun M, Kang JS, Sachid AB, Desai SB, Hettick M, Hu CC, Javey A (2014). Field-effect transistors built from all two-dimensional material components. ACS Nano.

[246] Späh R, Lux-Steiner M, Obergfell M, Bucher E, Wagner S (1985). n-MoSe_2_/p-WSe_2_ heterojunctions. Appl. Phys. Lett..

[247] Yu JH, Lee HR, Hong SS, Kong D, Lee H-W, Wang H, Xiong F, Wang S, Cui Y (2015). Vertical heterostructure of two-dimensional MoS_2_ and WSe_2_ with vertically aligned layers. Nano Lett..

[248] Li M-Y, Shi Y, Cheng C-C, Lu L-S, Lin Y-C (2015). Epitaxial growth of a monolayer WSe_2_/MoS_2_ lateral p-n junction with an atomically sharp interface. Science.

[249] Lee C-H, Lee G-H, van der Zande AM, Chen W, Li Y (2014). Atomically thin p–n junctions with van der Waals heterointerfaces. Nat. Nanotechnol..

[250] Nourbakhsh A, Zubair A, Dresselhaus MS, Palacios T (2016). Transport properties of a MoS_2_/WSe_2_ heterojunction transistor and its potential for application. Nano Lett..

[251] Cheng R, Li D, Zhou H, Wang C, Yin A (2014). Electroluminescence and photocurrent generation from atomically sharp WSe_2_/MoS_2_ heterojunction p–n diodes. Nano Lett..

[252] Jariwala D, Sangwan VK, Wu C-C, Prabhumirashi PL, Geier ML, Marks TJ, Lauhon LJ, Hersam MC (2013). Gate-tunable carbon nanotube–MoS_2_ heterojunction p-n diode. Proc. Natl. Acad. Sci..

[253] Chuang S, Kapadia R, Fang H, Chia Chang T, Yen W-C, Chueh Y-L, Javey A (2013). Near-ideal electrical properties of InAs/WSe_2_ van der Waals heterojunction diodes. Appl. Phys. Lett..

[254] Zhang W, Huang Z, Zhang W, Li Y (2014). Two-dimensional semiconductors with possible high room temperature mobility. Nano Res..

